# Effects of Propolis Extract and Propolis-Derived Compounds on Obesity and Diabetes: Knowledge from Cellular and Animal Models

**DOI:** 10.3390/molecules24234394

**Published:** 2019-12-01

**Authors:** Hiroshi Kitamura

**Affiliations:** Laboratory of Veterinary Physiology, School of Veterinary Medicine, Rakuno Gakuen University, Ebetsu 069-8501, Japan; ktmr@rakuno.ac.jp; Tel.: +81-11-388-4781

**Keywords:** propolis, bee product, metabolic disorder, type 2 diabetes, oxidative stress

## Abstract

Propolis is a natural product resulting from the mixing of bee secretions with botanical exudates. Since propolis is rich in flavonoids and cinnamic acid derivatives, the application of propolis extracts has been tried in therapies against cancer, inflammation, and metabolic diseases. As metabolic diseases develop relatively slowly in patients, the therapeutic effects of propolis in humans should be evaluated over long periods of time. Moreover, several factors such as medical history, genetic inheritance, and living environment should be taken into consideration in human studies. Animal models, especially mice and rats, have some advantages, as genetic and microbiological variables can be controlled. On the other hand, cellular models allow the investigation of detailed molecular events evoked by propolis and derivative compounds. Taking advantage of animal and cellular models, accumulating evidence suggests that propolis extracts have therapeutic effects on obesity by controlling adipogenesis, adipokine secretion, food intake, and energy expenditure. Studies in animal and cellular models have also indicated that propolis modulates oxidative stress, the accumulation of advanced glycation end products (AGEs), and adipose tissue inflammation, all of which contribute to insulin resistance or defects in insulin secretion. Consequently, propolis treatment may mitigate diabetic complications such as nephropathy, retinopathy, foot ulcers, and non-alcoholic fatty liver disease. This review describes the beneficial effects of propolis on metabolic disorders.

## 1. Introduction

The global prevalence of obesity brings about increased incidence of various metabolic diseases such as adipocity and type 2 diabetes mellitus (T2DM). In particular, the number of patients suffering from T2DM has dramatically increased during the last several decades [1,2]. In the Global Report on Diabetes, the World Health Organization predicted that the number of patients with diabetes will increase to seven billion by 2025. Thus, establishment of cost-effective therapeutics for T2DM is assumed to bring large profits to governments [3]. T2DM is the consequence of insulin resistance or insufficient insulin secretion [4]. Prolonged hyperglycemia causes glycation of proteins and lipids by enzyme-independent reactions, leading to the generation of advanced glycation end products (AGEs) [5]. Ligation of AGEs to the receptor for advanced glycation end products (RAGE) activates nuclear factor-κB (NF-κB) [6,7], which potentiates the production of reactive oxygen species (ROS) [5,7]. High glucose also stimulates ROS production in the mitochondria of glucose sensitive cells, such as islet β-cells and endothelial cells [8,9,10]. In addition to eliciting apoptosis, ROS provoke the upregulation of cytokines, such as interleukin (IL)-1β and tumor necrosis factor-α (TNF-α), via NF-κB activation, which subsequently exacerbates local inflammation [10]. ROS-induced local inflammation contributes to the development and progression of diabetic complications such as nephropathy, retinopathy, and non-alcoholic fatty liver disease (NAFLD) [11,12,13,14]. Moreover, promotion of lipolysis in adipocytes causes elevated circulation of very low density lipoprotein-cholesterol (VLDL-C) and low density lipoprotein-cholesterol (LDL-C), both of which are assumed to be key determinants of the pathogenesis of atherosclerosis [15]. Subsequently, excess amounts of lipids are deposited in hepatocytes, resulting in the development of NAFLD [16,17]. Therefore, type 2 diabetes is believed to be one of the major exacerbating factors of NAFLD and of a consequent disease, non-alcoholic steatohepatitis (NASH) [18]. Indeed, 30–50% of patients with type 2 diabetes experience NAFLD [19]. Furthermore, an epidemiological study conducted over the past three decades has indicated that obesity and type 2 diabetes are major predictors of NAFLD [20].

Propolis, a hive product, is a mixture of honeybee saliva, extracts from seeds and leaves, and exudates of plant flora. So far, more than 500 chemical compounds have been isolated from propolis [21]. Since the major ingredients of propolis are derived from plants, the chemical composition of propolis is highly dependent on its geographical origin. In general, propolis can be classified into two types: the poplar type, which is abundant in flavonoids and the Baccharis type, which is abundant in cinnamic acid derivatives [21,22,23]. Poplar type propolis is mainly produced in Europe, North America, and the non-tropical regions of Asia, while the Baccharis type propolis is produced in Brazil [24]. Propolis has been used in folk medicine and complementary therapies to treat a wide variety of diseases [25,26]. On the other hand, scientific verification has demonstrated that propolis and its chemical components have actual therapeutic effects on infectious disease, inflammation, and cancer [26,27,28]. Moreover, accumulating evidence indicates that propolis also has beneficial effects for metabolic disorders. In this review, I summarize the effects of propolis and propolis-derived chemical compounds on obesity, diabetes, and complications resulting from diabetes. Since type 1 diabetes mellitus (T1DM) and T2DM cause similar complications due to hyperglycemia, I also include effects of propolis on T1DM. In this review, particular focus is given to data obtained using animal and cellular models, which provide knowledge about the cellular and molecular mechanisms underlying the effects of propolis.

## 2. Advantages and Disadvantages of Animal and Cellular Models

### 2.1. Animal Models

To consider mechanisms underlying the progression of metabolic complications, crosstalk between organs cannot be ignored. From this point of view, experimental animal models have an apparent advantage over cellular models. In addition, precise knowledge about drug metabolism and kinetics cannot be acquired through in vitro models. Among various animal models, rodents, especially mice and rats, are most frequently used for animal experiments because of their biological characteristics. The litter size of mice is 6–13, while the average litter size of rats is 10 [29,30]. Moreover, mice rapidly reach sexual maturity (less than 8 weeks after birth) and have a relatively short reproductive cycle and gestation period (~3 weeks) [29]. The large litter size allows researchers to obtain many biological replicates while maintaining a similar genetic background. Short life spans (mouse, 2 years; rat, 2–3 years) are another merit to using these rodent models to investigate metabolic disorders. Most metabolic diseases such as T2DM and NAFLD gradually progress in humans over several decades. Taking advantage of the short life span of these animals, data about the pathogenic processes of metabolic disorders can be obtained over a relatively short duration (several months to one year) [29,31]. Small body size is also a merit, especially with regards to experimental cost. The small size of these experimental animals allows downsizing of breeding space, injected drug volumes, and waste. Additionally, mice and rats can be microbiologically (e.g., specific pathogen free and germ-free animals) and genetically controlled in experimental animal facilities. Thus, a limited number of individuals can provide reproducible and reliable data. Furthermore, several experimental intervention protocols, such as caloric control, surgical and chemical treatments, and gene manipulation, have been developed for mice and rats [31,32]. Accordingly, several protocols and assay kits for the assessment of metabolic disorders are well established [33]. Therefore, experiments using rodents are a global standard in drug validation for metabolic disorders.

Biological differences between humans and rodents should be taken into account for animal experiments. Indeed, there are numerous differences in behavior, energy metabolism, mucosal flora, and the immune system, all of which critically affect metabolic states [29,34]. For instance, mice feed at all hours, while human usually fast through the night. Thus, fasting glucose and insulin levels are absent in mice under normal physiological conditions. As a consequence, the effects of fasting on insulin sensitivity might be different between humans and mice [34]. The thermal effects on energy metabolism are also quite different between mice and humans [35]. Thus, the researchers should understand biological characteristics of animal models, and should select models for their purpose. Since streptozotocin (STZ)-induced diabetic mice have severe destruction of pancreatic islets, the mice cannot be used to study insulin resistance in T2DM [36]. On the other hand, STZ-treated mice have provided plenty of knowledge about glucotoxicity, which is a critical issue for T2DM. Likewise, although leptin-deficient monogenic mutant rodents show obesity and diabetes immediately after ablation [29], human T2DM is most prevalent in the middle aged and elderly. Thus, the data obtained using these models cannot be simply applied to human therapies. Nevertheless, these animals are useful models to investigate molecular and cellular events in obese and diabetic individuals, and they provide knowledge about the effects of anti-obesity or anti-diabetes agents in several tissues at an individual level [33]. 

Regarding studies of propolis or propolis-derived compounds, differences among species in pharmacokinetics and pharmacodynamics should be taken into consideration. Since several factors such as genotype, age, sex, habitual diet, medications, and gut microbiota are known to affect metabolism of flavonoids and polyphenols even among human individuals [37,38]; the capacity for metabolism of the compounds is likely to be quite different between human and animal models. Of the cytochromes P450 (CYPs), which are the main enzymes involved in metabolizing chemical compounds, CYP1A, 2C, 2D, and 3A have different catalytic activities in different mammalian species, such as humans, mice, and rats [39]. Given that CYP1A and CYP2C contribute to the metabolism of dietary flavonoids and polyphenols [40,41], the kinetics of propolis-derived compounds in animal models are likely to be different from those in humans. Similarly, interspecies differences in intestinal microbiomes have been exhibited between mice and humans, and these differences severely affect the various metabolic profiles of polyphenols [38]. Transplantation of human microbiota into gnotobiotic mice might be a way to overcome the microbiome-derived differences in metabolism of propolis-derived compounds [38]. On the other hand, especially for diabetes and obesity research, one should also consider the adverse effects of a long-lasting burden of excess calorie intake on the metabolic capacity of hepatocytes. Indeed, a high fat diet (HFD) decreased oxysterol detoxification due to aberrant activities of CYPs in mice [42]. Thus, the kinetics of chemical compounds in propolis might be determined by a sum of species-specific factors and damage to hepatocytes by excess calorie burden in animal models suffering from adiposity and T2DM. Therefore, effective doses of propolis or propolis-derived compounds in animal studies might not be directly applicable to human clinical studies.

To perform animal experiments, researchers should employ proper protocols. The intent of the Animal Research: Reporting of In Vivo Experiments (ARRIVE) guidelines is to improve transparency in reporting research using animals [43]. The purpose of ARRIVE is to obtain the most effective data using the smallest number of animals [43]. Recently, several major scientific journals have claimed to collect experimental data based on ARRIVE [44]. Thus, scientists should design animal experiments satisfying the guidelines for ethical issues and scientific soundness according to ARRIVE.

### 2.2. Cellular Models

Despite the lack of information about multicellular interactions, in vitro cellular models are usually employed to clarify cellular events directly evoked by propolis and propolis-derived compounds. For example, the propolis-derived compound chrysin abrogates diabetic nephropathy in obese mice [45,46,47,48]. Progression of diabetic nephropathy is attributed to the dysfunction of various types of renal cells, such as glomerular epithelial cells, podocytes, and mesangial cells [45,46,47,48]. The dissection of the direct effects of chrysin on each renal cell type is required to understand the consequences of cellular events evoked in the kidney by chrysin treatment. Moreover, experiments using a single or limited number of cell types provide knowledge about detailed molecular events directly evoked by propolis or propolis-derived compounds. Indeed, by employing adipocyte cell lines, one can identify kinases and transcription factors whose activity is directly modified by propolis supplementation [49,50,51].

## 3. Obesity and Adipocity

### 3.1. Experimental Models

#### 3.1.1. Animal Models and Pathology

One of the explicit phenotypes induced by excessive caloric intake is obesity. Obese rodent models have conventionally been generated using an HFD, composed of 45–60 kcal% fat, for several months [31,33]. In the case of a 60 kcal% fat HFD, overweight phenotype appeared after 2 weeks; at 4–5 months, mice reached weights 20–30% heavier than mice fed with normal chow diet [31]. The diet-induced obese (DIO) mice also exhibited several forms of metabolic dysfunction owing to insulin resistance [29,31]. Among inbred mice, C57BL/6 mice have been well employed since they develop severe adiposity more easily [52]. Genetically obese animal models are also often used for obesity research. Truncated non-functional leptin-expressing ob/ob mice and inactive leptin receptor-expressing db/db mice present with hyperphagia and obesity immediately after ablactation [31,53,54]. Similarly, Zucker fatty rats have a missense mutation in the leptin receptor gene, resulting in substitution of glutamine for proline at the 269th amino acid from the N-terminal [55]. Zucker fatty rats show significant increase in body weight by five weeks of age, and their obesity progressively deteriorates with age [56].

In addition to body weight, the wet tissue weight of adipose tissue is often used as an index of obesity. White adipose tissues are roughly classified into visceral and subcutaneous adipose tissues. According to human etiology, visceral adipose tissue weight is highly correlated with metabolic diseases such as type 2 diabetes, hypertension, and dyslipidemia [57]. In mice, visceral adipose tissues include epicardial, perigonadal, mesenteric, perirenal, and retroperitoneal adipose tissues, each of which show functional heterogeneity [58]. To increase adipose tissue mass, adipocytes undergo hypertrophy (an increase in size) and hyperplasia (an increase in number). Large lipid-laden adipocytes in hypertrophic tissue in particular prefer to secrete several mediators that trigger metabolic dysfunction [59]. Thus, histological observation of adipocytes is one of the approaches to evaluate the pathological staging of obesity-associated diseases. In parallel with adipocyte hypertrophy, the infiltration of immune cells such as macrophages into adipose tissue is frequent [60]. Infiltration of immune cells causes mild inflammation in the adipose tissue [60,61]. Adipose tissue inflammation increases circulating levels of adipocytokines, fatty acid mediators (lipokines), and miRNA-containing exosomes, all of which affect energy metabolism in the liver and skeletal muscle [62,63,64]. Thus, circulating levels of adipocyte-derived substances is another diagnostic signature for obesity from a pathological perspective [63]. Leptin is an atypical adipokine dominantly produced in hypertrophic adipocytes [65,66]. Leptin is transported across the blood brain barrier via a saturable transporter system [67] and function as a satiety signal for the arcuate nucleus of the hypothalamus [68]. Leptin also regulates energy expenditure by controlling activity of the sympathetic nervous system [69]. Chronically obese individuals acquire leptin resistance and show deterioration of energy metabolism [70]. Adiponectin is another popular adipokine that promotes fatty acid oxidization and glucose utilization in the liver and skeletal muscle through activation of AMP-activated protein kinase (AMPK) [71,72]. Moreover, adiponectin promotes fat combustion [73]. By contrast, TNF-α, which is secreted by adipose tissue macrophages [74], deteriorates insulin sensitivity via inhibition of insulin receptor (IR) tyrosine kinase activity in adipose and muscle tissue [75,76,77]. In addition to inflammatory cytokines, several secretion products from hyperplastic adipocytes also promote metabolic disorders. Plasminogen activator inhibitor (PAI)-1, as its name indicates, functions as an inhibitor of tissue plasminogen activator and also of urokinase, and is secreted from adipocytes into the circulatory system [78]. PAI-1 participates in thrombosis of obesity-induced ischemic stroke [79], and has a direct casual roles in obesity and insulin resistance [80]. 

Progression of obesity is dependent on three factors: energy intake, deposit, and expenditure. Energy intake is determined by feeding, and thus it is one of the therapeutic targets of anti-obesity medications. In this context, periodic monitoring of body weight and food intake in model animals is an essential process in the exploration of appetite-repressing drug candidates. The brain’s “feeding center” in the lateral hypothalamus, neuropeptide Y (NPY)-producing neurons in the arcuate nucleus, the “satiety center” in the ventromedial hypothalamus, and proopiomelanocortin-producing neurons in the arcuate nucleus all directly and indirectly accept inputs from the vagus nerve, peptide hormones, and metabolites [81,82,83]. Thus, modulation of these afferent signals is proposed to prevent obesity.

In healthy human individuals, approximately 60% of energy is consumed by the skeletal muscle, liver, and brain as basal metabolism [84]. Although basal metabolic rate can be increased by exercise, effective medications potentiating energy metabolism in these organs are difficult to develop. On the other hand, activation of brown adipocytes and their relatives, beige adipocytes, has been extensively attempted. Both brown and beige adipocytes express uncoupling protein 1 (UCP1), which produces heat by leaking protons from the mitochondrial inner membrane [85]. Thus, brown and beige adipocytes consume but do not deposit energy. Additionally, brown/beige adipocytes have the potential to improve glucose and lipid metabolism by UCP1-independent mechanisms, including the secretion of “batokines” [86]. Previous papers have demonstrated that peroxisome proliferator-activated receptor γ (PPARγ), PPARγ coactivator 1α (PGC1α), and PR-domain-containing 16 (PRDM16) are crucial factors to elicit brown/beige adipocyte differentiation [87]. In other words, activation of a complex constituted by these factors might promote energy expenditure leading to prevention of obesity. Recently, some papers have tried to assess whether propolis-derived compounds induce UCP1-positive cells in mice [88,89].

#### 3.1.2. Cellular Models

To evaluate mechanisms of differentiation and the cellular function of adipocytes, mouse-derived 3T3-L1 cells are most widely used. 3T3-L1 preadipocytes differentiate into mature adipocytes in the presence of a 3-isobutyl-1-methylxanthine, dexamethasone, and insulin (MDI) cocktail [90]. MDI stimulates induction of PPARγ and CCAAT/enhancer-binding protein (C/EBP)α, resulting in induction of mature adipocyte signatures, such as fatty acid binding protein 4 (FABP4)/adipocyte protein 2 (aP2) [91]. To achieve complete differentiation, the differentiation medium is often supplemented with rosiglitazone (2 μM) [92]. After 7–10 days in the differentiation medium, the cells accumulate lipid droplets, which can be checked by Oil Red O or Sudan II staining [93,94,95]. At this time, β-adrenergic stimulation increases cAMP, and subsequently activates protein kinase A (PKA) in differentiated 3T3-L1 cells [96]. Eventually, PKA activates hormone sensitive lipase (HSL) via phosphorylation of Ser-659 and Ser-660 of HSL, consequently leading lipolysis [97]. On the other hand, acute treatment of insulin inhibits activation of HSL and promotes glucose uptake through inducing translocation of glucose transporter (GLUT) 4 from the cytoplasm in 3T3-L1 cells [98,99]. Mature 3T3-L1 cells also produce several adipokines, such as leptin, adiponectin, resistin, TNF-α, and IL-6 [100]. Therefore, 3T3-L1 cells can be used to investigate effects of propolis and its derivative components on the endocrine function of hypertrophic adipocytes.

Excess intake of carbohydrates and lipids results in their deposition as triglyceride in adipocytes. Adipose tissue adapts to hyperlipidemia through hypertrophy and hyperplasia [59]. As previously mentioned, hypertrophic adipocytes secrete especially harmful adipokines; scientists have made great efforts to find substances to direct adipocytes toward hyperplasia rather than hypertrophy [59]. Nuclear receptor PPARγ stimulates de novo differentiation of adipocytes and apoptosis of lipid-laden adipocytes, resulting in miniaturization of adipocytes in adipose tissue [101]. PPARγ binds fat-soluble substances, such as 15-deoxy-∆12,14-prostagrandin J2, oxidized LDL, and long chain fatty acids [102,103,104]. Subsequently, PPARγ forms a heterodimeric complex with retinoic acid X receptor (RXR). This ligand-PPARγ-RXR complex binds to the peroxisome proliferator response element (PPRE) and promotes differentiation of preadipocytes into adiponectin-producing small adipocytes [105]. In addition, PPARγ also modulates inflammatory signals through interaction with NF-κB [106], suggesting that a PPARγ agonist could suppress low-grade inflammation of adipose tissue in obese individuals. Therefore, putative PPARγ ligands discovered in the natural ingredients of propolis are being explored as therapies for obesity-associated diseases. 

### 3.2. Effects of Propolis and Propolis-Derived Components on Obesity

#### 3.2.1. Body Weight and Adipose Tissue Weight

To date, plenty of studies have examined whether propolis affects the body weight and adipose tissue weight of obese animals (Table 1). Ichi et al. reported that eight weeks of feeding with pellets containing 0.5% (*w*/*w*) Brazilian propolis did not affect body weight gain in rats [107]. The propolis diet did repress the weight gain of mesenteric, perirenal, and total white adipose tissues but not epididymal white adipose tissue in obese rats [107]. In the HFD-induced obese rats, propolis down-regulated PPARγ protein levels in parallel with a decrease of white adipose weight [107]. Hence, propolis might regulate adipose tissue hypertrophy via repression of PPARγ. On the other hand, there is another report demonstrating that feeding supplemented with Brazilian propolis extract (50 mg/kg/day) for 10 days by stomach intubation significantly repressed visceral adipose tissue weight as well as body weight gain of HFD-fed C57BL/6N mice [108]. The same report also showed that 25 mg/kg propolis ethanol extract attenuated body-weight gain in pre-existing obese mice [108]. In this experimental condition, only perirenal adipose tissue had decreased wet tissue weight, whereas neither retroperitoneal nor parametrial adipose tissues were modulated [108]. Anti-adipocity effects were also observed in a monogenic mutant obese model. We demonstrated that repeated intraperitoneal injection of Brazilian propolis extracts (100 mg/kg, twice a week for 12 weeks) significantly decreased mesenteric adipose tissue mass, whereas weights of epididymal and inguinal adipose tissue was not modulated [109]. These previous reports indicated that effects of propolis were different between adipose tissues. In general, visceral adipose tissue is more sensitive to propolis than subcutaneous adipose tissue. Propolis produced in other geographical locations also has anti-obesity effects. For example, oral administration of Croatian propolis (ethanol extract, 50 mg/kg/day) decreased body weight gain of C57BL/6N mice after 10–20 days of treatment [110]. Accordingly, the anti-obesity effects of propolis-derived chemicals have also been investigated. Caffeic acid phenethyl ester (CAPE, 0.02–0.5%), a hydroxycinnamic acid in propolis, significantly decreased body weight gain in HFD-fed C57BL/6N mice in a dose-dependent manner [111]. Moreover, CAPE decreased epididymal adipose tissue mass without affecting kidney and liver weights [111]. Thus, CAPE seems to be one of the compounds responsible for the anti-obesity effects of propolis.

#### 3.2.2. Dyslipidemia

The advantageous effects that propolis has on lipid profiles in obese mice and rats has also been well documented. Rats fed Brazilian propolis-containing pellets (0.05% and 0.5%) displayed inhibition of increase in plasma triglycerides and total cholesterol in a dose-dependent manner [107]. Moreover, two doses (42.5 and 425 mg/kg) of propolis inhibited the increase of circulating triglyceride after feeding with olive oil (5 mg/kg) [107]. On the other hand, oral administration of Croatian propolis ethanol extract (50 mg/kg/day) for 30 days decreased serum triglycerides (~11%), total cholesterol (~19%), and LDL-C (~35%) levels in DIO C57BL/6N mice, while the level of high density lipoprotein-cholesterol (HDL-C) was not modified [110]. The propolis-treated mice showed improved atherogenic indices of plasma, atherogenic coefficients, cardiac risk ratios, and cardioprotective index, all calculated from the lipid profile parameters [110]. Roquetto et al. investigated effects of a 0.2% crude Brazilian propolis–containing diet on the blood lipid indices of DIO C57BL/6 mice [112]. They observed that propolis treatment significantly decreased blood triglyceride levels compared to the control HFD group, although it failed to decrease blood total cholesterol and HDL-C levels. Koya-Miyata et al. reported that intragastric injection of Brazilian propolis (5 mg/kg for 10 days) significantly attenuated triglyceride, cholesterol, and non-esterified free fatty acid (NEFA) levels in HFD mice [108]. They also reported that 2.5 mg/kg propolis mitigated serum NEFA increase and tended to reduce serum triglyceride levels in the DIO model [108]. The therapeutic effects of Brazilian green propolis on dyslipidemia in *ob/ob* mice were also assessed [109]. Intraperitoneal injections of propolis ethanol extract (100 mg/kg, twice per week for 12 weeks) slightly decreased total cholesterol levels of *ob/ob* mice while not affecting triglyceride nor NEFAs levels [109]. Collectively, propolis has the potential to normalize dyslipidemia, although collectively, previous reports have indicated variability in its effects.

#### 3.2.3. Feeding and Leptin Production

An in vitro study using 3T3-L1 adipocytes showed that Brazilian green propolis ethanol extract (100 μg/mL) upregulated leptin expression (Table 2) [113]. Considering the anorectic activity of leptin, propolis has potential to attenuate feeding and subsequently preventing obesity. In agreement, intraperitoneal injection of Brazilian green propolis ethanol extract (100 mg/kg, twice per week for 12 weeks) strongly repressed feeding of C57BL/6 mice, accompanied by a two-fold increase in leptin expression in the epididymal adipose tissue [113]. Given that the same treatment with propolis extract in *ob/ob* mice failed to modulate feeding [109], leptin is responsible for the anorexic effects of intraperitoneal injections of propolis extract. In contrast to intraperitoneal injection, oral supplementation with Brazilian propolis extract did not modulate food intake in mice and rats [107]. Therefore, one or more leptin-inducing substances in Brazilian propolis do not seem to reach significant levels in blood circulation after oral supplementation, presumably due to degradation by gastric acid, malabsorption by intestinal epithelial cells, rapid metabolism in the liver, or discharge as urine. So far, there is one report describing the effects of CAPE on leptin expression in 3T3-L1 adipocytes [114]. In that report, 3T3-L1 cells were treated with different doses (0, 10, 25, and 50 μM) of CAPE during the last five days of differentiation [114]. The leptin expression level was suppressed by CAPE in a dose-dependent manner [114]. Concomitantly, CAPE-treated 3T3-L1 cells showed a down-regulation of insulin receptor substrate-1 (IRS-1), which is a prerequisite for adipocyte differentiation [115]. Thus, the CAPE-induced decrement of leptin in 3T3-L1 cells seems to be attributed to insufficient differentiation. More recently, Vanella et al. assessed the effects of CAPE (10 μM) on leptin expression in mature adipocytes that were differentiated from adipose stem cells (ASCs) isolated from human subcutaneous adipose tissue [116]. They observed that CAPE remarkably attenuated leptin expression in ASCs-derived adipocytes, accompanied by a decrement of lipid droplets [116]. Thus, Brazilian propolis is likely to contain unknown substance(s) capable of inducing leptin, surpassing the repressive effects of CAPE on leptin expression.

#### 3.2.4. Adipogenesis

**Figure 1:**
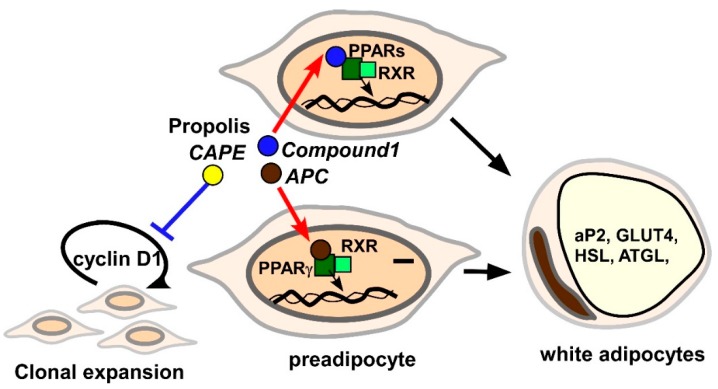
Effects of propolis-derived compounds on adipogenesis. Chrysin derivative compound **1** and artepillin C (APC) stimulate differentiation of adipocytes in a PPAR/RXR-dependent manner. On the other hand, caffeic acid phenethyl ester (CAPE) represses clonal expansion by disturbing the ERK/Akt-cyclin D1 cascade.

So far, several lines of study have demonstrated that propolis and derived chemical compounds modulate adipogenesis in cell culture systems (Figure 1). 

The direction of effects on adipogenesis is dependent on the substances employed for the analyses. For instance, an ethanol extract of Brazilian red propolis (20–30 μg/mL) promotes differentiation of post-confluent 3T3-L1 preadipocytes in accordance with induction of mature adipocyte signatures, such as aP2, GLUT4, HSL, adipose triglyceride lipase (ATGL) and adiponectin [117]. A reporter analysis suggested that PPARγ participates in the adipogenic effects of Brazilian red propolis extracts [117]. Artepillin C (APC) is a *Baccharis dracunculifolia*-derived compound, which is present in Brazilian propolis. A molecular docking analysis identified APC as a direct ligand for PPARγ [118]. Similar to a chemical PPARγ ligand, rosiglitazone, APC (10 μM) stimulated accumulation of lipid droplets in 3T3-L1 cells [49]. In addition, the PPARγ-selective inhibitor GW9662 inhibited APC-elicited adipocyte differentiation in a dose-dependent manner [49]. APC (10 μM or 25 μM) induced mature adipocyte markers, such as aP2 and GLUT4, which were dampened by GW9662 treatment [49,118]. Interestingly, 20 μM APC did not increase but rather decreased leptin mRNA in mature 3T3-L1 cells [113]. Hence, one or more other distinct components seem to be involved in leptin induction. Additionally, compound **1** (8-[1-(4′-hydroxy-3′-methoxyphenyl)prop-2-en-1-yl]-chrysin), which was originally isolated from Mexican propolis [119], also functions as a ligand for PPARα, γ and δ, and promotes differentiation of human bone marrow mesenchymal stem cell (hBM-MSC)-derived adipocytes at a 10 μM dosage [51]. In contrast to APC and compound **1**, CAPE was found to suppress differentiation of adipocytes. For instance, 25–50 μM CAPE decreased triglyceride deposition in 3T3-L1 cells after stimulation with MDI [120]. Furthermore, CAPE (10–50 μM) treatment significantly repressed induction of PPAR-γ, C/EBP-α, aP2, and fatty acid synthase, as well as glyceraldehyde-3-phosphate dehydrogenase (GAPDH) activity in 3T3-L1 cells [114,120]. The molecular mechanisms underlying CAPE-induced impaired expression of adipocyte signatures in adipocytes have been also investigated. 3T3-L1 cells treated with 40 μM CAPE remained at the G1/S checkpoint even after treatment with MDI [111]. CAPE blocked phosphorylation of ERK and Akt and led to failure to increase cyclin D in 3T3-L1 cells in response to the differentiation stimulus [98]. Therefore, CAPE is likely to inhibit activation of the Akt/ERK-cyclin D axis, resulting in aberrant adipogenesis. A trial was previously conducted to generate novel artificial CAPE analogues [121]. Some compounds markedly inhibited MDI-induced adipogenesis of 3T3-L1 cells [121]. Since the compounds inhibit pancreatic lipase activity and absorption of triglyceride from the intestine, they might become more effective anti-obesity materials than CAPE [121]. 

#### 3.2.5. Adipokine Production

As previously mentioned, Brazilian green propolis ethanolic extract and CAPE positively and negatively controlled leptin production in adipocytes, respectively [113,116]. In addition to leptin, propolis and propolis-derived chemicals also regulate production of several adipokines (Table 2). Adiponectin is known to be a beneficial adipokine secreted from small adipocytes [122]. Brazilian red propolis ethanol extract (20 μg/mL, 3 days) induced adiponectin mRNA in post-confluent 3T3-L1 preadipocytes, possibly through activation of the adiponectin promoter by PPARγ [117]. Moreover, Brazilian red propolis extract (5 and 10 μg/mL, 8 days) also restored adiponectin expression in TNF-α-treated, differentiated 3T3-L1 cells [117]. There have also been reports about chemicals upregulating adiponectin in adipocytes. For example, APC (10 or 25 μM) potentiated adiponectin expression by 1.5–2.0-fold in 3T3-L1 cells [49,118]. Similarly, compound **1** (10 μM) also potentiated adiponectin secretion in hBM-MCS-derived adipocytes [51]. Although CAPE (10 μM) decreased leptin expression, it evoked a more than two-fold increase in adiponectin expression in human ASC-derived adipocytes [116]. Collectively, several compounds in propolis have been found to positively regulate adiponectin expression in adipocytes.

Regarding harmful adipokines, CAPE (10 μM) down-regulated TNF-α, IL-1β, IL-6, and IL-8 expression in ASC-derived adipocytes [116]. Suppressive effects of CAPE on TNF-α were also evident in differentiated 3T3-L1 cells, although a higher dose (50 μM) was required [114]. CAPE (25 or 50 μM) also decreased both mRNA and intracellular protein levels of resistin in 3T3-L1 cells [114]. Considering that both TNF-α and resistin confer insulin resistance, CAPE seems to interfere with obesity-induced insulin resistance. So far, therapeutic effects of propolis on circulating PAI-1 levels elevated in obesity have not been documented. However, a diet containing 0.5% Brazilian propolis for 8 weeks decreased bacterial lipopolysaccharide (LPS)-induced blood PAI-1 by half [123]. Given that blood LPS level is elevated in obesity [112], propolis might modulate PAI-1 production in the adipocytes of obese individuals.

#### 3.2.6. Induction of Brown/Beige Adipocytes

**Figure 2:**
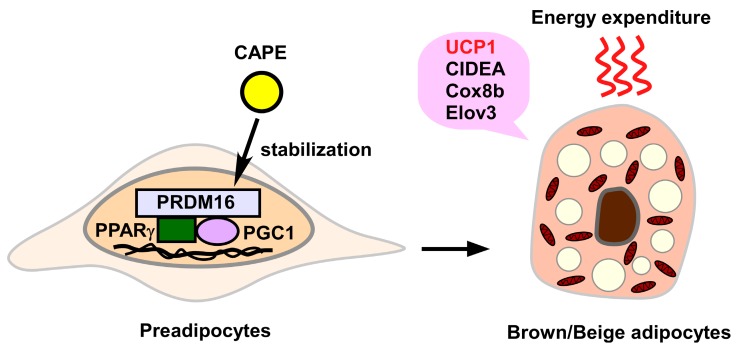
Effects of caffeic acid phenethyl ester (CAPE) on brown/beige adipocyte differentiation. CAPE stabilizes PR-domain-containing 16 (PRDM16). Consequently, supplementation with CAPE stimulates preadipocytes to differentiate into the brown/beige adipocytes, which express Uncoupling protein **1** (UCP1), a prerequisite mitochondrial protein for heat production.

In addition to influencing white adipocytes, propolis-derived chemical compounds are able to affect brown/beige adipocytes (Figure 2).

Nishikawa et al. reported that APC (1–10 μM) induced brown adipocyte markers, including uncoupling protein **1** (UCP1), cell death inducing DFFA like effector A (CIDEA), cytochrome C oxidase subunit VIIIb (Cox8b), and elongation of very long chain fatty acids-like 3 protein (Elovl3) in adipocyte-like C3H10T1/2 cells and primary cultured adipocytes isolated from the inguinal adipose tissue of C57BL/6J mice [88]. In this case, APC stabilized the transcription factor PRDM16, leading to the increased levels of intracellular PRDM16 [88]. Accordingly, oral administration of APC (5 or 10 mg/kg/day for 4 weeks) significantly increased the number of UCP1-expressing cells in inguinal adipose tissue, indicating that APC promotes induction of beige adipocytes [88]. Since repeated administration of APC did not increase norepinephrine content in inguinal adipose tissue, the sympathetic nervous system seems to be dispensable for the APC-elicited “beiging” of white adipocytes [88]. Recently, the same group also demonstrated that APC and curcumin, a yellow pigment of turmeric, synergistically promoted induction of brown-like adipocytes in the inguinal adipose tissue [89]. Therefore, a combined diet containing APC and curcumin can effectively attenuate adiposity through induction of brown(-like) adipocytes in white adipose tissue.

## 4. Diabetes Mellitus

### 4.1. Experimental Models

#### 4.1.1. Animal Models and Pathology

So far, various animal models have been employed to investigate the effects of propolis on diabetes mellitus (Table 3). To investigate the effects of propolis on diabetes, streptozotocin (STZ)-induced rodent models of diabetes have been most widely used because of convenience, effectiveness, and cost performance. STZ, which was originally isolated from Streptomyces achromogenes, specifically causes necrosis of pancreatic β-cells [36,124,125]. Two days after a single intraperitoneal injection of STZ (60–200 mg/kg), rats and mice exhibit severe hyperglycemia followed by diabetic complications [36,124]. Because of the selective destruction of β cells, STZ-induced models of diabetes are considered to mimic human T1DM [124]. Alloxan, a toxic glucose analog, is used to eliminate islet β-cells by promoting the accumulation of intracellular reactive oxygen species (ROS) [125]. S961 is an insulin receptor (IR) antagonist peptide that has also been used to block insulin signaling in animal models [126,127,128].

To investigate T2DM, which accounts for more than 90% of patients with diabetes, several experimental models have been established. A 40–60 kcal% fat HFD, either alone or in combination with a glucose or sodium chloride diet, is considered to mimic Western diet-induced human T2DM [29]. C57BL/6 strains are widely used with the DIO model since their pathological phenotype progresses quickly and dramatically [29,52,167]. The T2DM mouse models display low-grade inflammation in the adipose tissue [168]. Four weeks after beginning the HFD, a crown-like structure consisting of dead adipocytes and inflammatory macrophages emerges in the white adipose tissue [169]. The density of the crown-like structure gradually increases over 16 weeks [169]. Adipose tissue inflammation is believed to be the primary source of inflammatory cytokines, which affect insulin sensitivity of other tissues [168]. C57BL/6J mice exhibit increased blood glucose and insulin levels after one week on an HFD [167]. The blood insulin of the HFD-fed C57BL/6J mice progressively increased from baseline over 52 weeks [167]. Moreover, insulin and glucose tolerance were apparent after one and three weeks on the HFD, respectively [167]. Although C57BL/6N mice rapidly develop severe hyperglycemia, hyperinsulinemia, and subsequent hepatosteatosis after only three weeks on an HFD [170], pathogenesis progresses more slowly in other strains. Therefore, effects of overnutrition as well as aging should be taken into account for interpretation of experimental results, especially using other strains. To shorten the experimental period, a combinatory treatment of HFD with low dose STZ (for example, 40 mg/kg) has also been used [171].

Although monogenic T2DM is rare in human beings, genetic mutation models are also commonly employed for diabetes studies. Leptin and leptin receptor mutants, respectively the *ob*/*ob* and *db*/*db* mice, show severe diabetic manifestations, such as hyperglycemia and hyperinsulinemia within two weeks after birth [172,173]. Similarly, in Zucker fatty rats, serum insulin increases to a peak of 400 μU/mL at 15 weeks of age, whereas blood glucose is normal in both young and old rats [174]. Zucker Diabetic Fatty (ZDF) rats, a derivative of Zucker fatty rats, show severe diabetes in addition to obesity [175]. Cholecystokinin receptor A-deficient Otsuka Long-Evans Tokushima Fatty (OLETF) rats are another popular diabetes model [176]. They show hyperphagia in both light and dark phases [177] as well as spontaneous hyperglycemia after 18 weeks of age, followed by diabetic complications [178]. Goto-Kakizaki rats are a spontaneous diabetes model derived from Wistar rats [179]. Goto-Kakizaki rats have defects in glucose-stimulated insulin secretion from pancreatic β-cells, followed by diabetic nephropathy and retinopathy [180,181,182]. Since Goto-Kakizaki rats develop diabetes regardless of adipocity, the rats are considered to be a non-obese diabetes model [183]. Natural mutant models have critical issues regarding the identification of genes responsible for metabolic disorder, which is time consuming and labor-intensive. However, recent advances in DNA sequencing technologies have enabled the faster identification of the mutated loci responsible for T2DM progression [184]. On the other hand, gene editing technologies, such as clustered regularly short palindromic repeat (CRISPR)/CRISPR-associated protein 9 (Cas9) and transcription activator-like effectors (TALEs) also provide an opportunity to obtain novel genetic diabetes models using rodents and other vertebrates [184,185,186].

The most typical symptom of diabetic animal models is a high blood glucose level. Since blood glucose level is critically affected by feeding, fasting blood glucose (FBG) level is widely used to evaluate progression of diabetes. FBG levels of lean C57BL/6J mice are less than 100 mg/dL after an 12 h daytime fast, while those of eight week-HFD obese C57BL/6J mice increased to 150 mg/dL [187]. The major reason for high FBG is insulin resistance. In insulin resistant individuals, high levels of fasting blood insulin are also observed owing to the activation of a positive feedback loop [188]. The homeostasis model assessment of insulin resistance (HOMA-IR) is an index that enables us to determine insulin resistance by simple calculations [189]. To improve the accuracy of the evaluation, insulin tolerance is also widely assessed during primary screening tests [34]. In this assay, blood glucose levels are periodically measured after an intraperitoneal injection of recombinant human insulin [34]. The dose of insulin should be selected depending on experimental design: comparison between relatively lean mice is performed using a relatively low dose of insulin (e.g., 1.0 U/kg), whereas a higher dose of insulin (e.g., 1.5–5.0 U/kg) is required for obese models such as DIO and *ob*/*ob* mice [109,190]. High FBG also occurs when there is insufficient secretion of insulin from islet β-cells. Accordingly, the glucose tolerance test, in which blood glucose is measured after glucose administration, is performed to investigate the total defects in insulin secretion and insulin sensitivity. A previous paper indicated that oral administration of 2 g/kg of glucose following a six hour fast was optimal to evaluate glucose tolerance for C57BL/6J mice [191]. By performing both insulin and glucose tolerance tests, the glucose sensitivity of islet cells can be elucidated. For secondary assessments, the glucose clamp technique can be conducted to qualify insulin secretion and resistance. Glucose clamp techniques can be classified into hyperglycemic and hyperinsulinemic-euglycemic. The hyperglycemic clamp technique evaluates the sum of insulin secretion and glucose metabolism, while the hyperinsulinemic-euglycemic clamp technique measures insulin sensitivity [34].

Prolonged exposure to high level glucose causes glycation of proteins by non-enzymatic processes. Sugars such as glucose, fructose, and galactose, which have an aldehyde residue, react with a thiol- or amino-residue of a protein, resulting in the formation of a Schiff base [186]. Subsequently, the Schiff bases form Amadori products [192]. Glycated haemoglobin and albumin, atypical Amadori products, are widely used in the diagnosis of diabetes [193,194]. Since the average maximum life span of erythrocytes is 117 ± 12 days, glycation of haemoglobin is dependent on blood glucose level for a relatively short period [195]. Indeed, haemoglobin1Ac (HbA1c) is believed to reflect blood glucose level for three months [196]. On the other hand, glycated albumin can be used to assess blood glucose level for a shorter period since its half-life is less than three weeks [197]. For animal models, Hb1Ac is often used to assess diabetic states [129,130,131,154,157].

In diabetic conditions, ROS are produced in various tissues by the malactivation of mitochondrial electron transport, nonenzymatic glycation, glucose autoxidation, and NADPH oxidase (Nox) activation [8,198]. Oxidative damage by ROS is believed to be a primary cellular event in islet β-cells of T2DM patients. Since β-cells abundantly express GLUT2, hyperglycemia facilitates mitochondrial respiration, leading to excess accumulation of ROS in β-cells [198]. Moreover, β-cells are easily damaged by ROS, owing to their low expression levels of catalase (CAT) and glutathione peroxidase (GPx), both of which are present at only ~5% of the levels seen in liver cells [8]. As a consequence, β-cells decrease insulin production through several ROS-elicited mechanisms, and cell death follows [198]. Oxidative stress and oxidative damage are also observed in various tissues, including the liver, kidney, and brain of diabetic animals [199,200,201]. Thus, the antioxidation abilities of tissues can be tested to elucidate intrinsic anti-diabetes properties. For this purpose, amounts of antioxidant enzymes such as GPx, CAT, superoxide dismutase (SOD), glutathione S-transferase (GST), and antioxidant glutathione (GSH) have often been measured [131,132,133,134,135,136,137,138,139,140,155,157,162,202]. Alternatively, oxidation of polyunsaturated fatty acids is also measured to evaluate oxidative stress in diabetic animals. ROS degrade polyunsaturated fatty acids, resulting in the formation of malondialdehyde (MDA) [203]. Hence, accumulation of MDA is a useful index of oxidative stress [131,133,135,136,137,138,139,155,157]. To quantify tissue MDA content, a simple colormetric 2-thiobarbituric acid reactive substances (TBARS) assay is usually performed [203].

Hyperglycemia provokes excess accumulation of intracellular glucose in various cells, including endothelial cells, resulting in overproduction of mitochondrial ROS [204]. Overabundant ROS cause DNA damage, resulting in the activation of poly(ADP-ribose) polymerase (PARP) [204]. Activated PARP ADP-ribosylates GAPDH, which is a key enzyme in the glycolytic pathway [205]. ADP-ribosylated GAPDH has lowered enzymatic activity, resulting in the accumulation of glyceraldehyde 3-phosphate (G3P) [205,206]. Subsequently, G3P is converted to methylglyoxal, which is the major precursor in the formation of advanced glycation end products (AGEs) [207]. Moreover, glycated protein-derived Amadori products are also known as a source of AGEs [208]. Thus, several pathways contribute to the production of AGEs during diabetes. Ligation of the AGE-receptor for advanced glycation end products (RAGE) triggers NF-κB activation, leading to further production of ROS [209]. Moreover, NF-κB is a crucial transcription factor for the production of proinflammatory molecules, including cytokines, chemokines, prostanoids, and nitric oxide [210]. Hence, RAGE activation elicits local inflammation and tissue damage [211]. RAGE are dominantly expressed in monocytes/macrophages, neural cells, kidney cells, and vascular endothelial and smooth muscle cells [212]. Therefore, the AGE-RAGE ligation is believed to participate in glucotoxicity in blood vessels, and consequent pathological complications, such as diabetic nephropathy, retinopathy, and neural disease [213]. Therefore, the influence of propolis and its derived chemicals in AGE-elicited cellular signaling is a major topic in the apimedical field. Several reports have evaluated the effects of AGE on target tissue function in animal models [46,214,215].

Numerous reports in the literature have demonstrated that mild inflammation in visceral adipose tissue triggers insulin tolerance. Lipid-laden adipocytes secrete extremely high levels of metabolic disorder-associated factors, such as TNF-α, IL-6, resistin, and leptin [216]. In particular, hyperleptinemia causes leptin tolerance, which can exacerbate insulin resistance [217]. Progression of adipose tissue inflammation is controlled by immune cells in the tissue. Macrophages are the most abundant cell population in adipose tissue. Macrophages can be roughly classified by function into the M1 and M2 types. Classical M1 macrophages accelerate inflammatory responses through secretion of proinflammatory cytokines, while alternatively, active M2 macrophages maintain local homeostasis through tissue remodeling and anti-inflammatory effects [218]. In the adipose tissue of a lean individual, M2 macrophages are the dominant type of macrophages present. An excessive accumulation of lipid droplets results in adipocyte cell death and the formation of a crown-like structure follows, in which activated M1 macrophages phagocytose the debris of dead adipocytes [169,218]. Activated M1 macrophages in adipose tissue aggravate metabolic disorder through the secretion of several inflammatory molecules, such as TNF-α and IL-1β [219]. Macrophage phenotypes, such as M1 and M2 macrophages, can be identified by their patterns of surface markers, gene expression, and cytokine production [220]. To evaluate macrophage states in specimens from mouse models, flow cytometry and quantitative reverse transcription-PCR (qRT-PCR) analyses are typically performed [109,221]. Inducible nitric oxide (iNOS) and proinflammatory cytokines, such as TNF-α and IL-6, are often used as markers for M1 macrophages, whereas CD206, CD163, and arginase 1 are known to be atypical M2 macrophage markers for FACS and qRT-PCR analyses [103,220,221,222]. On the other hand, researchers should take into account that macrophage phenotypes show high plasticity and become altered dynamically depending on the microenvironment [220]. In addition to macrophages, there are several types of immune cells that modulate the inflammatory state of adipose tissue. Regulatory T cells, Th2 cells, eosinophils, type 2 innate lymphoid cells, and invariant natural killer T (iNKT) cells inhibit adipose tissue inflammation, while Th17 cells, Th1 cells, CD8^+^ T-lymphocytes, NK cells, neutrophils, and mast cells aggravate it [223]. Our lab has previously focused on the roles of CD11b^+^, Gr-1^+^ myeloid derived immune suppressor cells (MDSCs), which exclusively secrete immunosuppressive cytokine IL-10 in propolis-modulated adipose tissue inflammation [164]. Since the mass of visceral adipose tissues is well-correlated with progression of type 2 diabetes in etiological studies [57], epidydimal and mesenteric adipose tissue have been dominantly used for the assessment of the consequences of adipose tissue inflammation [109,221,224]. 

#### 4.1.2. Cellular Models

Since diabetes results in multi-organ defects, a myriad of models has been utilized to understand the pathogenesis of each complication. Circulating levels of glucose and other nutrients are highly dependent on biochemical responses in the liver. Thus, cultured hepatocyte-like models have been employed to investigate the molecular mechanisms underlying insulin tolerance. Isolated hepatocytes may be a desirable model in which to investigate the effects of propolis on hepatocytes. However, hepatoma cell lines, such as HepG2 cells, have previously been used [158,225,226], possibly owing to the ease of operation. After ligation of insulin and IR, IR β-chain (IRβ) is phosphorylated. Subsequently, IR substrate-1 (IRS-1), phosphatidylinositol-3 kinase (PI3K), 3-phosphoinositide-dependent protein kinase 1 (PDK-1), and Akt are activated sequentially [227]. Akt inhibits glycogenolysis as well as gluconeogenesis by several molecular mechanisms, including nuclear export of forkhead box class O1 (FoxO1) [228]. Thus, insulin-sensitivity is conventionally evaluated by the abundance ratio of the phosphorylated forms of IRβ, IRS-1, and Akt to the total amount of these proteins using specific antibodies [221]. Phosphorylation of glycogen synthase 3β (GSK3β) is another major index of insulin sensitivity, since GSK3 regulates glycogen synthase and IRS-1 [229]. Moreover, the activity and expression of key enzymes participating in glucose metabolism (e.g., glucose-6-phosphatase, hexokinase, and pyruvate kinase) have also been measured to determine the target’s reaction to propolis and its derivative compounds [158,226]. In cultured hepatocytes, palmitate increases ROS production in mitochondria and Nox3, leading to activation of c-Jun N-terminal kinase (JNK) and p38 MAP kinase [230,231]. Since gene silencing of Nox3 restored insulin sensitivity of hepatocytes, ROS seems to be a deteriorative factor for hepatic insulin resistance [231]. Therefore, elimination of oxidative stress is a possible way to overcome insulin resistance of hepatocytes.

Skeletal muscle is believed to have the predominant role in glucose utilization in both insulin-dependent and independent conditions [232]. On the other hand, skeletal muscle provides glucogenic amino acids, such as alanine and glycine, that are utilized in gluconeogenesis in the liver [233]. Thus, skeletal muscle is one of the determinants of blood glucose level. GLUT4 has essential roles in insulin-dependent glucose uptake in skeletal muscle [234]. After insulin stimulation, GLUT4 translocates from the cytoplasmic vesicle to the plasma membrane by activation of the PI3K/Akt pathway [235]. Moreover, AMPK enhances insulin-stimulated GLUT4 translocation [235]. Thus, compounds augmenting PI3K or AMPK activation might be used as anti-diabetic drugs. To elucidate biomedical effects of propolis on skeletal muscle in vitro, rat-derived L6 cells and mouse-derived C2C12 cells have been used [236,237]. 

Insulin secretion from β-cells is another determinant of diabetes mellitus. To evaluate whether propolis potentiates β-cell function, one study employed NIT-1 cells, which are generated from mouse islet cells with introduced SV40 large-T antigen [238]. Using NIT-1 cells, molecular mechanisms underlying glucose- and/or palmitate-induced impaired insulin secretion and apoptosis of β-cells have been extensively investigated [239,240,241]. Yuang et al. reported that high glucose concentration resulted in elevated Nox2 expression in NIT-1 cells accompanied by an increased production of ROS [242]. They also demonstrated that high levels of glucose activate JNK in a phosphatase and tensin homolog deleted from chromosome 10 (PTEN)-dependent manner. Consequently, a decrement of production and secretion of insulin occurred, possibly via the nuclear translocation of FoxO1 and cytoplasmic translocation of pancreatic duodenal homeobox-1 (PDX-1) [242].

Inflammation is a disease that underlies diabetic complications. Low grade inflammation of adipose tissue is a triggering event for type 2 diabetes [61,168]. Moreover, local inflammation contributes to the aggravation of diabetic nephropathy, retinopathy, and skin attrition [12,243,244]. Macrophages are pivotal cell populations that determine progression or alleviation of inflammatory responses through the secretion of pro- or anti-inflammatory cytokines [60]. In addition to metabolic disorders, macrophages are also associated with various diseases, including infectious disease, auto-immune disease, cognitive impairment, and cancer [245,246,247]. Hence, the biological effects of propolis and propolis-derived compounds on macrophages have been extensively studied using macrophages isolated from the peritoneal cavity or bone marrow [117,248,249] as well as macrophage cell lines such as RAW264 and J774.1 [117,164,250,251]. A large portion of the studies on the effects of propolis on macrophages focus on antioxidative stress and cytokine production [248,250,251], some of which seem to be applicable to anti-diabetic treatments. 

### 4.2. Effects of Propolis and Propolis-Derived Compounds on Diabetes Mellitus

#### 4.2.1. Blood Glucose, Hb1Ac, and Lipid Profiles

A large number of papers about the beneficial effects of propolis describe blood glucose content and lipid profiles. Alloxan treatment significantly increased blood glucose in SD rats at one week, and the potentiation continued to 8 weeks [202]. Accordingly, alloxan induced increment of FBG was restored by gavage of water (50 or 100 mg/kg/day) or ethanol (5 or 10 mg/kg/day) extracts of Chinese propolis [139]. Moreover, both extracts restored alloxan-induced changes in total cholesterol, LDL-C, VLDL-C, and HDL-C [139]. Likewise, oral administration of Nigerian propolis (200 or 300 mg/kg/day) decreased FBG level at two weeks, and partially decreased HbA1c at six weeks in alloxan-pretreated rats [154,155]. In this condition, Nigerian propolis treatment increased blood HDL-C, while it decreased blood LDL-C and cholesterol [154]. Beneficial effects on blood glucose level were also reported in STZ-treated mice and rats. Chinese propolis extract (100 or 200 mg/kg/twice daily for 12 weeks) in gum tragacanth slightly but significantly decreased FBG and Hb1Ac in STZ-pretreated rats [129]. Oral administration of Chinese and Brazilian propolis (100 mg/kg twice a day for 8 weeks) decreased blood glucose level one (Chinese) or three weeks (Brazilian) after treatment [130]. Concomitantly, HbA1c levels were significantly decreased in the Chinese propolis-treated group, while Brazilian propolis showed a trend toward decreasing levels [130]. In this report, while Chinese propolis did not, Brazilian propolis slightly (~17%) decreased total cholesterol levels, whereas propolis did not influence blood triglyceride, LDL-C, nor HDL-C levels [130]. Similar effects on blood glucose were also observed in propolis collected from Malaysia, Mexico, Iran, Egypt, Saudi Arabia, Turkey, and Croatia, although botanical sources are different [133,134,136,141,142,143,156,202]. Using S961 peptide as an inducer of T1DM, Indonesian propolis (50–200 mg/kg) lowered blood glucose level in a dose-dependent manner [128]. In addition to corrective effects, preventive effects of propolis on diabetes have been also reported. Matsushige et al. reported that pretreatment with the water extract of Brazilian Yukari propolis (200 mg/kg/day i.g. for 1 week) significantly repressed the STZ-induced FBG increase, while the same dose of methanol extract failed to modulate it [144].

The restoration of blood glucose and lipid profiles by propolis was also observed in T2DM models. A high fat-and high fructose-diet followed by an injection of low-dose STZ caused severe hyperglycemia, especially after one month [159]. Taiwanese green propolis ethanol extract (~184 or ~920 mg/kg/day) ameliorated the elevation of FBG level in the rats [159]. Sonication-encapsulated Chinese propolis (50–200 mg/kg) also attenuated FBG in HFD and low-dose STZ-treated diabetic rats [160]. The encapsulated propolis also inhibited the increase of serum triglycerides but not the total and LDL-C cholesterol levels [160]. Studies in the monogenic T2DM model also showed therapeutic effects of propolis on blood indices. Aoi et al. reported that a 0.1% or 0.5% Brazilian propolis-containing diet for eight weeks significantly decreased blood glucose, although plasma free fatty acids (FFAs) and β-hydroxy butyrate were not altered in OLETF rats [165]. On the other hand, intraperitoneal injections of Brazilian green propolis ethanol extract (100 mg/kg, twice a week for 12 weeks) was found to cause a ~2/3-fold decrease in blood glucose levels [109]. 

Several mechanisms underlying the hypoglycemic effects of propolis have been studied. These studies have mainly suggested that glucose uptake is impaired by oxidative stress (see below). Previous papers have proposed additional mechanisms underlying the acceleration of glucose utilization in skeletal muscle. Translocation of GLUT4 to the plasma membrane in L6 myotubes was enhanced in response to 1 μg/mL of Brazilian propolis, resulting in an acceleration of 2-deoxy glucose (2DG) uptake [236]. Since wortmannin (a PI3K inhibitor), LY294002 (a PI3K inhibitor), and compound **C** (an AMPK inhibitor) clearly inhibited 2DG uptake and translocation of GLUT4, this suggested the involvement of PI3K and AMPK in the propolis-promoted GLUT4 translocation [236]. In agreement with the in vitro study, oral administration of propolis extract (250 mg/kg) significantly increased GLUT4 content in the plasma membrane of skeletal muscle of ICR mice and SD rats and improved glucose tolerance in ICR mice [236]. Another report described inhibition of gluconeogenesis by propolis in hepatocytes. In the presence of high glucose (25 mM), HepG2 cells exhibited increased levels of glucose-6-phosphatase (G6Pase), a key enzyme for gluconeogenesis [225]. Treatment with 12.5 or 25 μg/mL of Korean propolis decreased the activity and mRNA expression of G6Pase in HepG2 cells, suggesting that propolis has the potential to attenuate glucose release from hepatocytes [225]. Since inhibitors of PI3K and Akt did not modify propolis-elicited G6P down-regulation, PI3K and Akt are unlikely to be direct or indirect targets of propolis [225]. In contrast, propolis strongly prevented serine- and tyrosine-phosphorylation of GSK3-α and β [225], which maintain G6Pase gene expression [252]. Since antioxidant N-acetylcysteine (NAC) failed to reverse the repression of G6Pase expression by propolis, ROS were not involved in the suppressive roles of propolis in gluconeogenesis through G6P modulation in HepG2 cells [225].

The anti-hyperglycemic and anti-dyslipidemia effects of ingredients of propolis have been also investigated. Poplar is one of the major ingredients of Chinese propolis. Application of 50% poplar bud extracts (50 or 100 mg/kg for 4 weeks) resulted in decreased serum levels of FBG, glycated hemoglobin, glycated serum protein, total cholesterol, and LDL-C in HFD/STZ-treated Kunming mice, whereas it did not influence serum triglyceride level [157]. Regarding chemical compounds, APC, coumaric acid, and kaempferide (1 μM for 15 min) enhanced phosphorylation of AMPK and PI3K and translocation of GLUT4 to the plasma membrane of L6 cells [236]. Of the chemicals, kaempferide increased 2DG uptake in the cells [236]. Thus, these compounds might have the potential to improve the insulin sensitivity of skeletal muscle. On the other hand, chrysin attenuated hyperglycemia in T2DM model animals [161,163]. Feeding with an HFD for 90 days raised FBG to 160 mg/dL, while chrysin (25–100mg/kg/day for last 30 days) lowered the FBG level in a dose-dependent manner [163]. Concomitantly, chrysin also decreased serum TG, total cholesterol, LDL-C, VLDL-C, and FFAs, whereas HDL-C increased [163]. In accordance with these findings, chrysin (40 mg/kg/day) attenuated FBG to normal levels in HFD/STZ–treated T2DM rats [161]. T2DM is usually prevalent in aged individuals. Recently, intraperitoneal injections of chrysin (20 mg/kg/day for 4 weeks) were found to slightly decrease FBG, TG, total cholesterol, and LDL-C in aged Wister rats [253]. Additionally, therapeutic effects of chrysin (25 mg/kg/day p.o. for 8 weeks) as well as luteolin (100 mg/kg/day) on triglyceride, total cholesterol, and LDL-C were also reported in a T1DM model [254]. Quercetin, another flavonoid in propolis, is known to have therapeutic effects on diabetic model animals. Intraperitoneal injection of quercetin (15 mg/kg/day for 4 weeks) three days prior to STZ treatment repressed the increase in serum glucose level from 334 mg/dL to 132 mg/dL [145]. Moreover, CAPE (15 or 30 mg/kg p.o. for 5 weeks) also decreased FBG from 23.6 mmol/L to 12–15 mmol/L in HFD/STZ-treated C57BL/6J mice [158]. Both doses of CAPE also modulated serum total cholesterol, LDL-C, and HDL-C levels, although only the higher dose decreased total triglyceride levels. Pinocembrin (10 mg/kg i.g. for 40 days), a marker of poplar type propolis, also lowered blood glucose, total cholesterol, LDL-C, and triglyceride levels in STZ-treated Wister rats [146]. 

Another possible mechanism through which propolis could decrease blood glucose is decreased glucose adsorption from the intestine. Indeed, several flavonoids such as luteolin, amentoflavone, luteolin 7-*O*-glucoside, and daidzein have been found to strongly inhibit the activity of α-glucosidase, which is critical for glucose adsorption [255]. Moreover, an ex vivo study demonstrated that naringenin (500 μM) maintained the integrity of Na^+^-glucose symport in intestinal and renal membranes in STZ-treated diabetic rats [256]. Collectively, several chemical compounds in propolis have beneficial effects on blood glucose and lipid levels in diabetic and elderly patients.

#### 4.2.2. Blood Insulin Level, Insulin Secretion, and Insulin Resistance

**Figure 3:**
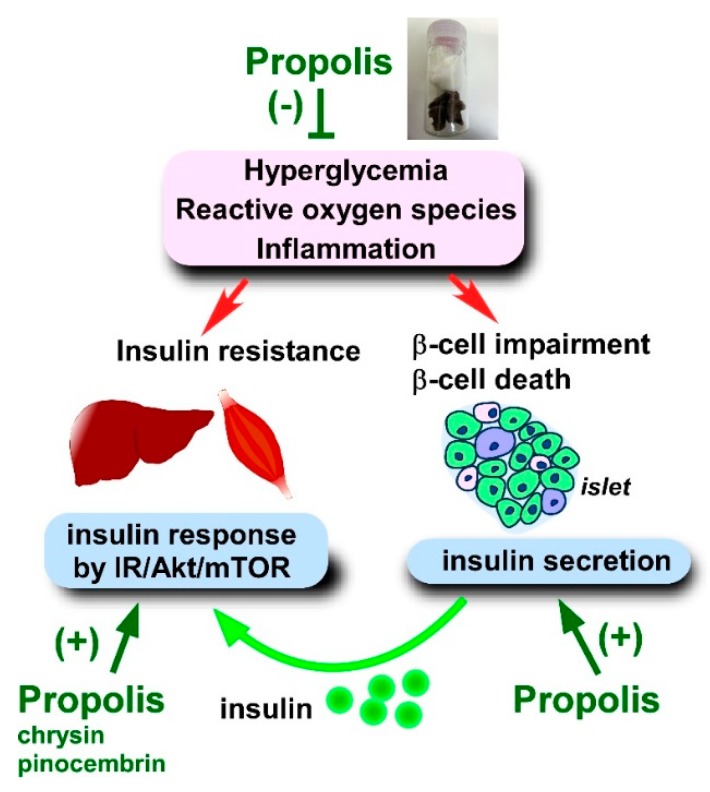
Restoration of insulin secretion and insulin signaling by propolis. Propolis prevents hyperglycemia, production of reactive oxygen species, and inflammation, all of which account for insulin resistance and islet β-cell dysfunction. Additionally, propolis potentiates insulin secretion from β-cells and also improves insulin sensitivity by modulating insulin receptor (IR)/Akt/mammalian target of rapamycin (mTOR) signaling.

To date, various reports have demonstrated that propolis and its derived compounds improve insulin secretion and insulin sensitivity using animal and cellular models (Figure 3). 

Several reports have assessed the effects of propolis on serum insulin level in STZ-induced diabetic models. Since STZ destroys pancreatic β-cells, blood insulin levels are remarkably decreased. An ethanol extract of Malaysian propolis (300 mg/kg/day) partially protected STZ-induced insulin deficiency [133]. Accordingly, Malaysian propolis improved HOMA-IR index, HOMA-β, insulin sensitivity, and pancreatic β-cell function [133]. Importantly, combinatory treatment of propolis and metformin completely abolished the defects of the insulin-related changes [133]. Similar data were obtained using ethanol extracts of Mexican and Saudi Arabian propolis [134,142]. Mexican propolis (300 mg/kg/day for 15 days) increased the detection of serum insulin in STZ-treated diabetic mice [135]. Additionally, the Mexican propolis also disrupted the elimination of islet β-cells by STZ. Thus, propolis is capable of preventing β-cell destruction through STZ-elicited mechanisms. Since many flavonoids scavenge ROS, flavonoids such as quercetin might protect β-cells from oxidative damage [135]. To support this idea, quercetin (15 mg/kg/day i.p. for 4 weeks) was found to partially recover the STZ-induced insulin deficiency in rats [145].

In addition to ROS removal in the pancreas, propolis affects the mechanism of insulin secretion. Pancreatic β-cells promote insulin secretion in response to arginine [257]. Arginine is postulated to interact with the arginine target for insulin secretion (AITS) complex in the endoplasmic reticulum of β-cells [257]. Brazilian propolis, but not Chinese propolis, mimics the effects of arginine on NIT-1 cells. Drupanin (10 μM) and APC (10 μM), both of which are present in *Baccahris dracunculifolia*, also cause insulin secretion from NIT-1 cells at a comparable level to arginine (100 μM) [257]. Of interest, Brazilian propolis (0.01%) had a more prominent effect on insulin secretion compared to arginine, drupanin, and APC, suggesting that Brazilian propolis involves unidentified stronger stimulants for insulin secretion [257]. The same authors also demonstrated that propolis administration increased the circulating insulin level in lean mice in concert with a decrement of blood glucose [257]. 

In the T2DM model, hyperglycemia is attributable to the sum of insulin insensitivity and impaired insulin secretion. Owing to insulin insensitivity, feedback mechanisms increased fasting insulin level, especially during the early phase of T2DM. A previous report indicated that a 0.5% Brazilian propolis ethanol extract-containing diet induced a ~78% decrease of plasma insulin in OLETF rats, suggesting recovery of insulin sensitivity [165]. To support this, we also showed that repeated injection of Brazilian green propolis (100 mg/kg, twice a week for 12 weeks) dramatically improved the insulin and glucose tolerance of *ob/ob* mice, although feeding and body weight were not affected [109]. A similar observation reported that the oral administration of Brazilian propolis (100 or 300 mg/kg for 4 weeks) ameliorated the serum insulin level and HOMA-IR in OLETF rats [166]. Improvement of insulin sensitivity was also reported following treatment with Chinese propolis. Li et al. reported that encapsulated Chinese propolis (50–200 mg/kg/day for 10 weeks) decreased fasting serum insulin (~56 μIU/mL to 40–44 μIU/mL) and insulin action index in T2DM model rats [160]. The euglycemic hyperinsulinemic glucose clamp test also showed a significant improvement of insulin sensitivity in the Chinese propolis (100 mg/kg)-treated group compared with the control group [160]. In accordance with these findings, poplar bud (50 or 100 mg/kg/day for 4 weeks), a main ingredient of Chinese propolis, caused a more than 25% decrease in serum insulin level of HFD- and STZ-induced T2DM model mice [157]. Of note, effects of both doses of poplar bud extract on serum insulin level were more prominent than that of metformin (100 mg/kg/day) [157]. 

Various chemical compounds in propolis modulate insulin sensitivity. CAPE (15 or 30 mg/kg for 5 weeks) significantly improved fasting insulin level, HOMA-IR, and glucose tolerance in HFD/STZ treated C57BL/6J mice [158]. The CAPE-treated mice displayed significantly decreased circulating TNF-α, IL-6, and monocyte chemoattractant protein-1 (MCP-1) [158]. Consistent with the idea that TNF-α worsens insulin sensitivity in the liver, both doses of CAPE increased phosphorylation of IRS and Akt in the liver, skeletal muscle, and epididymal adipose tissue of diabetic mice [158]. Since the higher dose of CAPE attenuated phosphorylation of JNK and nuclear localization of NF-κB, the JNK-NF-κB inflammatory signal seems to be an effective target of CAPE [158]. The same report also demonstrated that CAPE (60 ng/mL) ameliorated impairments of glucose consumption and glucose uptake, as well as changes in G6Pase and glycogen contents in the insulin-resistant HepG2 cells by impeding JNK-NF-κB signaling [158]. 

Restoration of the insulin response by chrysin has been also extensively investigated. For instance, chrysin (20 mg/kg/day i.p. for 4 weeks) rescued the age-related decrement of serum insulin level in Wister rats, suggesting that chrysin maintains islet function [253]. Similarly, oral administration of chrysin (100 mg/kg/day for 30 days) mitigated the aberrant contents of IR, IRS-1, and phosphorylated IRS-1 in the gastrocnemius muscle in HFD-induced T2DM model rats, suggesting an improvement in insulin signaling [163]. In sharp contrast, Liu et al. demonstrated that chrysin (0.5–4 μM) as well as pinobanksin (4–32 μM) did not modulate the insulin-induced glucose consumption of HepG2 cells, which were pre-treated with insulin (5 μM) for 36 h [226]. Unfortunately, the reasons for the discrepancies between the reports have not been clearly addressed.

In terms of other compounds, galangin (10–80 μM) and pinocembrin (1–4 μM) overcame the insulin resistance of the cells [226]. Both galangin and pinocembrin potentiated insulin-induced glycogen accumulation in the insulin resistant HepG2 cells, along with recovery of hexokinase and pyruvate kinase activities [226]. Galangin treatment (especially 80 μM) restored the aberrant phosphorylation of IR, IRS, Akt, GSK3α/β, mammalian target of rapamycin (mTOR), and ribosomal protein S6 (RPS6) in insulin resistant HepG2 cells, implying that galangin is a modulator of Akt/mTOR signaling [226]. Similarly, pinocembrin also seemed to target the IR/Akt/mTOR pathway: 4 μM of pinocembrin modified the phosphorylation of IR, IRS, PTEN, Akt, and GSKβ [226]. Given that the IR/Akt/mTOR pathway is a determinant of insulin sensitivity, galangin and pinocembrin are likely to abrogate insulin resistance in hepatocytes through modulation of the IR/Akt/mTOR pathway. 

#### 4.2.3. Oxidative Stress

Oxidative stress is a fundamental molecular event that causes cellular dysfunction in diabetic patients. Given that flavonoids and phenols are atypical natural antioxidants, a myriad of studies have supposed that flavonoids and phenols in propolis disturb the progression of diabetes through their antioxidative activities (Table 4). 

Intraperitoneal injection of water or ethanol extracts of Croatian propolis (50 mg/kg/day i.p. for 7 days) prevented body weight reduction by alloxan-induced diabetes [156]. In this situation, both extracts of Croatian propolis notably decreased MDA content in liver and kidney in accordance with histological restoration [156]. The same group also demonstrated that a water-soluble derivative of Croatian propolis (50 mg/kg/day for 7days) mitigated lipid peroxidation in the liver, kidney, brain, and spleen in alloxan-treated mice [146]. 

As with European propolis, the major plant origin of Chinese propolis is poplar. Similar to Croatian propolis, Chinese propolis presents antioxidative activity in in vivo models. Water (50 or 100 mg/kg/day i.g.) or ethanol (5 or 10 mg/kg/day i.g.) extracts of northern Chinese propolis significantly attenuated the blood level of fructosamine and MDA in alloxan-treated rats, whereas SOD levels were increased [202]. Chinese propolis (100 mg/kg i.g. for 8 weeks) also decreased blood and renal MDA levels in STZ-pretreated diabetic rats, although it did not affect hepatic MDA [130]. Since slight increases in blood SOD, renal CAT, and hepatic GPx were observed [130,131], alteration of the activities of these enzymes might be involved in the antioxidant activity of propolis. Accordingly, Chinese propolis (400 μg/mL) normalized the vascular reactions of high glucose (44 mM)-exposed rat aortas by decreasing TBRAS and increasing SOD [259]. Moreover, Chinese propolis ethanol extract (200 mg/kg/day for 12 weeks) reduced serum ROS and reactive nitrogen species (RNS) in STZ-pretreated diabetic rats [129]. 

Since Brazilian propolis is the Baccharis type, the antioxidative stress efficacy of Brazilian propolis is assumed to be different from European and Chinese propolis. So far, there are few papers comparing antioxidant effects of Brazilian and Chinese propolis extracts. Zhu et al. reported that Brazilian propolis (100 or 200 mg/kg/day for 8 weeks) has more prominent remedial effects on blood nitric oxide synthase (NOS), SOD, and MDA levels in STZ-treated rats compared with the same dose of Chinese propolis [130,131]. The same reports also indicated more beneficial effects of Brazilian propolis on hepatic SOD, GSH, and MDA and renal MDA in T1DM model rats [130,131]. Likewise, Brazilian propolis ethanol extract (100–300 mg/kg/day i.g. for 40 days) decreased serum as well as renal and pancreatic MDA, accompanied by increases in serum and renal SOD, GSH, and CAT and pancreatic SOD in STZ-pretreated rats [137,138].

There are a lot of studies in the literature showing propolis produced in other areas confers resistance to oxidative stress evoked by diabetes. Mexican propolis (300 mg/kg/day i.g. for 15 days), which includes naringin, naringenin, kaempferol, quercetin, acacetin, luteolin, pinocembrin, and chrysin, raised the activities of SOD, CAT, and GPx in the pancreas of STZ (130 mg/kg)-induced diabetic CD1 mice [134]. Similarly, treatment with Malaysian propolis (300 mg/kg/day) raised the activities of SOD, CAT, GPx, GSH, GST, glutathione reductase (GR), and total antioxidant activity in the pancreas of STZ-treated (60 mg/kg) rats, while MDA content was significantly decreased [133]. In agreement with the fact that ROS induces inflammation and apoptosis, the propolis-treated diabetic rats had decreased IL-1β, TNF-α, RelA, and cleaved caspase 3 in the pancreas [133]. The same authors demonstrated that Malaysian propolis also potentiated total antioxidant activity, possibly through activation of SOD, CAT, GPx, GST, GR, and GSH in the liver [133]. Protective effects on β-cells against oxidative stress have also been described with Taiwanese propolis ethanol extract (183.9 or 919.5 mg/kg/day i.g. for 8 weeks) in STZ/HFD model rats [159]. On the other hand, gavage of Iranian propolis (200 mg/kg/day for 6 weeks) recovered SOD, GPx, and ferric-reducing antioxidant power (FRAP) in the kidney of STZ-induced diabetic rats while it decreased renal MDA content [136]. Furthermore, Nigerian propolis (200 or 300 mg/kg/day for 28 days) restored blood antioxidant indices in the alloxan-treated rats [155]. Decreases of ROS were also evident in the bone marrow, spleen, blood, and liver of Saudi Arabian propolis (100 mg/kg/day for 1 month)-treated diabetic mice [142]. Additionally, a methanol extract of propolis, which was purchased from a local herbal medical shop in Saudi Arabia, also ameliorated disorders of antioxidant enzymes and MDA accumulation in the serum and kidney in STZ-induced diabetic rats [135]. These results collectively indicate that propolis blunts oxidative stress in animal models, regardless of the production area of propolis.

As expected, several chemical compounds in propolis could scavenge oxidative stress in diabetic animals (Table 5). 

Oral administration of the antioxidant flavonoid chrysin (100 mg/kg/day for 30 days) significantly decreased MDA, H_2_O_2_, and hydroxyl radicals in the muscles of HFD-induced T2DM model rats [163]. Likewise, chrysin (40 mg/kg/day for 16 weeks) led to the accumulation of total GSH, GPx, GR, SOD, and CAT, resulting in a decrease of MDA in the kidney of HFD/STZ-induced T2DM model rats [161]. Pinocembrin (10 mg/kg i.g. for 40 days), another flavonoid enriched in poplar, also attenuated plasma and renal MDA and urinary H_2_O_2_ in STZ-treated Wister rats [146]. As previously mentioned, quercetin has the capacity to scavenge ROS. Quercetin (15 mg/kg/day i.p. for 4 weeks) treatment abrogated the increase in erythrocyte MDA, serum NO, and pancreatic MDA, SOD, CAT, and GPx of STZ-treated rats [145]. On the other hand, the effects of CAPE on oxidative stress are somewhat controversial and differ between reports. Gong et al. reported that daily intraperitoneal injections of CAPE (10 μmol/kg i.p. for 4 weeks) decreased MDA, protein carbonyl, and nitric oxide (NO), whereas SOD and CAT activities and GSH content in the kidney were significantly elevated in high fat, sugar- and cadmium-fed diabetic mice [162]. In contrast, the same dose of CAPE decreased SOD and CAT in the heart of STZ-induced diabetic SD rats, although GPx was increased [139]. While CAPE caused the decrement of some antioxidative enzymes in this model, MDA content was decreased in the heart, implying that ROS were scavenged [162]. CAPE also attenuated STZ-induced oxidative stress in the pancreas. Oral treatments of a non-toxic dose of CAPE (30 mg/kg/day for 21 days) restored non-protein thiol groups (RSH), lipid hydroperoxide (LOOH), and RNS levels in the pancreas and plasma in STZ-induced diabetic rats in concert with the decrease of asymmetric NG,NG-dimethyl-L-arginine (ADMA), an endogenous nitric oxide synthase inhibitor [260]. The authors also demonstrated that CAPE restored expression of nuclear factor erythroid-derived 2 (Nrf2)-dependent expression of antioxidant enzymes, such as heme oxygenese-1(HO-1), dimethylarginine dimethylaminohydrolase (DDAH), and γ-glutamyl-cysteine ligase (GGCL) in the pancreas [260]. In sharp contrast, the CAPE-treated model had a significant decrease in nitric oxide synthase (iNOS) [260]. These expression changes of enzymes induced by CAPE likely perturb STZ-induced ROS/RNS accumulation in the pancreas, leading to the maintenance of pancreatic integrity. 

#### 4.2.4. Systemic Inflammation and Immune System

In diabetic patients, both systemic and local inflammation emerges, and the increase of circulating proinflammatory cytokines subsequently leads to the deterioration caused by the disease. So far, several reports have indicated that propolis ameliorates cytokine induction in diabetic animals. Gavage of Taiwanese green propolis (919.5 mg/kg/day for 8 weeks) decreased serum TNF-α, IL-1β, and IL-6, while a lower dose (183.9 mg/kg/day) caused milder responses in TNF-α and IL-1β levels [159]. Similarly, administration of Saudi Arabian and Egyptian propolis extracts also diminished the increase of circulating IL-6 in STZ-induced diabetic animals [135,140]. Flavonoids or phenols in propolis also modify circulating cytokine levels in vivo. For example, CAPE treatment (15 or 30 mg/kg/day i.g. for 5 weeks) significantly attenuated serum IL-6, TNF-α, and monocyte chemotactic proten-1 (MCP-1) levels compared with control STZ/HFD-treated mice [158]. On the other hand, administration of chrysin (40 mg/kg/day i.g. for 16 weeks) lowered circulating IL-1β and IL-6 almost to half the levels of the untreated HFD/STZ-induced diabetic model [161]. Therefore, propolis and derivative compounds inhibit systemic inflammation by diabetes. Additionally, chrysin (20 mg/kg/day i.p. for 30 days) was found to modulate serum IL-1β, IL-6, TNF-α, and IL-10 levels in 20 month old male rats [253]. Given that systemic inflammation is a factor in the deterioration that accompanies diabetes, propolis may prevent high susceptibility to T2DM in aged individuals. 

A Western-style diet remarkably modulates the intestinal environment, resulting in dysbiosis [147]. Dysbiosis facilitates the introduction of bacterial products such as bacterial LPS into blood circulation, and thus aggravates chronic inflammation, which is highly associated with metabolic disease [261]. Feeding with Brazilian propolis (0.2%)-containing HFD for 5 weeks clearly decreased serum LPS content compared to control HFD-fed mice [261]. Concomitantly, the propolis-containing diet also modulated changes in blood glucose, triglyceride, total cholesterol, uric acid, total protein, and albumin [112]. Meanwhile, the propolis-containing diet significantly attenuated LPS-TLR4 signaling in the muscle: propolis supplementation decreased the protein abundance levels of TLR4, CD14, myeloid differentiation 2 (MD2), myeloid differentiation primary response 88 (MyD88), interleukin-1 receptor-associated kinase-1 (IRAK-1), and TNF receptor associated factor 6 (TRAF6) in muscle [112]. In agreement with these findings, propolis supplementation clearly decreased proinflammatory cytokines in muscle [112]. Since inflammatory cytokines confer insulin resistance to muscle [76], propolis seems to improve insulin sensitivity of the muscle. Accordingly, propolis-containing HFD restored growth of *Proteobacteria* in the ceca to the level of the normal diet group, although *Firmicutes* and *Thermotogae* were significantly decreased [112]. While *Bacteroidetes* tended to be decreased in the HFD-fed group, it returned to control-diet levels after propolis administration [112]. Hence, propolis ameliorates metabolic disorder through normalization of intestinal flora. In a similar context, propolis recovered metabolic dysfunction through suppression of periodontitis [262]. Oral administration of *Porphyromonas gingivalis*, an atypical periodontopathic bacterium, significantly upregulated TNF-α in the epididymal adipose tissue and tended to increase IL-6 in the liver, indicating the occurrence of low-grade systemic inflammation [262]. In accordance with these findings, *P. gingocalis* upregulated expression of lipid droplet formation- and gluconeogenesis-associated molecules (i.e., perilipin-2, acyl-CoA-reductase, and glucose-6-phosphatase) in the liver and downregulated expression of insulin sensitivity regulators (IRS1, sirtuin-1, and complement 1q and TNF related 9) in adipose tissue [262]. Meanwhile, an ethanol extract of Brazilian propolis (200 mg/kg/day p.o. for 5 weeks) dampened *P. gingocalis*-induced expressional changes in cytokine and metabolism-associated molecules, although it failed to abrogate periodontitis-associated alveolar bone loss [262]. Taken together with the more than 70% decrement in blood endotoxin activity induced by propolis treatment [262], propolis mitigates metabolic dysfunction by decreasing circulating bacterial product-induced inflammation.

Oršolić et al. proposed another possible effect of propolis on peritoneal macrophages. They showed that Croatian propolis (50 mg/kg/day for 7 days) promoted macrophage adherence capacity, which is an index for activated macrophages, in alloxan-induced diabetic mice [258]. Since propolis increased apoptotic and necrotic cells in the kidney of diabetic mice, the authors proposed tissue remodeling by propolis-activated macrophages [258].

There are also reports showing changes in characteristics of lymphocytes in diabetic model animals. For instance, oral supplementation with Saudi Arabian propolis extracts (100 mg/kg/day for 1 month) resulted in increased levels of cytokines involved in the proliferation of lymphocytes, such as IL-2, IL-7, and IL-4 in the plasma of STZ-pretreated mice, whereas it decreased proinflammatory cytokines such as IL-1β, IL-6, TNF-α [142]. Accordingly, the propolis-treated mice restored proliferation capacity of CD45R^+^, B220^+^ B lymphocytes, and CD45R^-^, B220^-^ T lymphocytes after IL-4- and CD40L-stimulation [142]. Moreover, propolis treatment also restituted CCL21- or C-X-C motif ligand (CXCL) 21-elicited chemotaxis of the isolated lymphocytes [142]. Collectively, propolis may prevent inflammation by maintenance of lymphocyte integrity. 

Rifa’i and Widodo further investigated the effects of Indonesian propolis on the profile of T-lymphocytes in the spleen of S961-pretreated Balb/c mice [128]. Propolis treatment (200 mg/kg i.g. for 14 days) apparently decreased CD8^+^, CD62L^+^ naïve T-lymphocytes from ~61 to ~9% in normal mice. Conversely, propolis administration lead to a slight increase of these cells, from ~24 to ~29%, in the diabetic mice [128]. A different response between healthy and diabetic mice was also observed in apoptotic T-lymphocytes in the spleen: propolis potentiated the proportion of apoptotic cells in the spleen by >4-fold in normal mice, while in diabetic mice it elicited remarkably limited changes in apoptosis (~2.3% compared to ~2.5%) [128]. Thus, these data suggest that alteration of sensitivity to propolis might occur in the lymphocytes in diabetic animals, although the physiological significance of this effect remains elusive. The authors also reported effects of propolis on the number of splenic regulatory T cells (Tregs), which repress immune activation via secretion of IL-10 and transforming growth factor (TGF)-β [263]. Propolis supplementation caused a >5-fold decrease in the number of splenic Tregs in diabetic mice, while in healthy mice it raised the abundance of Tregs by >5-fold [128]. Taken together, these findings indicate that propolis attenuates excess immune activation through a decrement of naïve cells and an increase of immune suppressive cells. 

#### 4.2.5. Adipose Tissue Inflammation 

**Figure 4:**
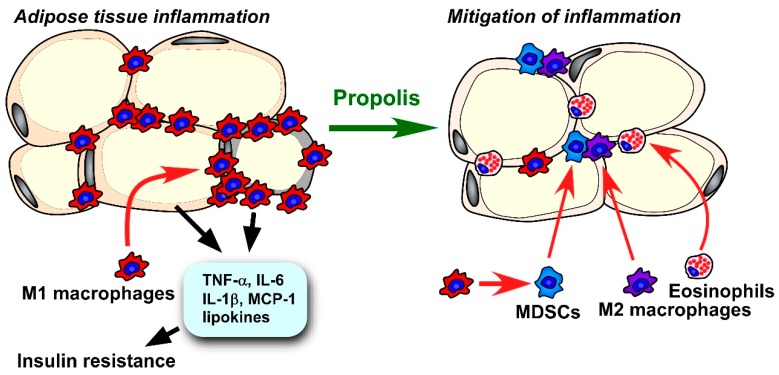
Effects of propolis on adipose tissue inflammation. In obese animals, M1 macrophages accumulate in visceral adipose tissues, followed by inflammation of adipose tissue. Several cytokines and lipokines produced in inflamed adipose tissue yield systemic insulin resistance. Propolis mitigates adipose tissue inflammation via accumulation of M2 macrophages, myeloid derived immune suppressor cells (MDSCs), and eosinophils.

Adipose tissue inflammation is a hallmark of the early phase of T2DM and contributes to the increase of circulating harmful cytokines (Figure 4) [60]. 

Intraperitoneal injection of Brazilian green propolis ethanol extracts (100 mg/kg i.p. twice a week for 12 weeks) prevented hyperplasia of the mesenteric adipose tissue of *ob/ob* mice [109]. Transcriptomics of the vascular stromal fraction of the visceral adipose tissue indicated that propolis significantly attenuated expression of immune-associated gene sets [109]. In agreement with this, the expression of M1 macrophage markers, such as *Itgax, Il12a, Il12b* and *Cd40*, was clearly decreased in the propolis-treated group, whereas expression of M2 macrophage markers, such as *Retnla, Irf4,* and *Tm7sf4*, was decreased [109]. Moreover, the propolis-treated mice manifested a dramatic decrement of the M1/M2 macrophage ratio and also indicated an increased number of eosinophils in the mesenteric adipose tissue [109]. Collectively, these results indicate that Brazilian propolis extract abates adipose tissue inflammation. Since eosinophils are considered to be a key suppressor of adipose tissue inflammation [264], eosinophils might be one of the initial targets of Brazilian propolis ethanol extract. 

In addition to eosinophils and M2 macrophages, CD11b^+^, Gr-1^+^ MDSCs were also increased in the visceral adipose tissues after intraperitoneal injection of Brazilian green propolis ethanol extract (100 mg/kg i.p. twice a week for 1 month) [164]. Induction of MDSCs in the visceral adipose tissue was observed not only in *ob/ob* or DIO mice, but also in lean C57BL/6J mice [164]. Moreover, propolis extracts also induced MDSCs in the peritoneum of mice. Importantly, direct treatment with propolis ethanol extract (100 μg/mL for 24 h) induced the MDSC marker *Ly6g,* as well as immune suppressive cytokine IL-10 in the bone marrow-derived M1 macrophages, peritoneal macrophages, and macrophage-like J774.1 cells [164]. Therefore, propolis is capable of promoting the transdifferentiation of inflammatory macrophages to inflammation-suppressive MDSCs in a direct manner. Among twelve flavonoids and natural phenols in Brazilian propolis, kaempferol (110 ng/mL) and pinocembrin (37 ng/mL) induced MDSCs from macrophages from J774.1 cells [164]. In agreement with this, kaempferol (10 mg/kg i.p. for 2 months) also yielded an accumulation of MDSCs in the epididymal adipose tissue of C57BL/6 mice [164]. Thus, kaempferol may be the compound in Brazilian propolis responsible for the amelioration of adipose tissue inflammation. 

#### 4.2.6. Vascular Endothelial Cells and Contraction of Vessels

Although vascular endothelial cells are the primary target of hyperglycemia, a limited number of papers have proven direct effects of propolis and its derivative chemicals on endothelial cells of diabetic models. El-Awady et al. reported that after incubation in high glucose (44 mM) medium the rat aorta ring showed weakened phenylephrine-induced contraction and acetylcholine-induced relaxation [259]. In this setting, nitroprusside, a NO donor, induced vasorelaxation to the same degree in both high and low (11 mM) glucose conditions, suggesting that endothelial cells are the dominant target of high glucose [259]. Chinese propolis ethanol extract (400 μg/mL) corrected the impairment of both contraction and relaxation of the aorta in response to high glucose treatment [255]. Thus, propolis seems to maintain endothelial integrity in hyperglycemic conditions. Promotion of vasorelaxation was also reported by chrysin (25 mg/kg/day) or luteolin (100 mg/kg/day) treated T1DM model rats [254]. Both compounds prompted acetylcholine-induced relaxation of the thoracic aorta of STZ-treated rats [254]. In this setting, impairment of NO generation in the diabetic rats was significantly recovered after luteolin not but chrysin treatment [254]. Thus, luteolin may enhance vasorelaxation via efficient NO production. On the other hand, the molecular mechanisms underlying the improvement of vascular relaxation caused by chrysin remain obscure. 

Borriello et al. assessed the effects of pinocembrin on endothelial cell death evoked by glycated insulin, an atypical AGE [265]. Pinocembrin (40 μM) nearly diminished glycated insulin-induced intracellular ROS accumulation in the bovine-derived CPAE endothelial cells, followed by inhibition of the nuclear translocation of NF-κB [265]. Pinocembrin also notably attenuated AGE-induced activation of caspases 3 and 7 [265]. Therefore, pinocembrin protected against endothelial cell death due to AGE exposure, possibly through the reduction of ROS, NF-κB, and/or caspase signaling.

Although this data was not obtained in high glucose conditions, the promotion of vascular regeneration by propolis suggests its therapeutic potential for circulatory failure in diabetic patients. In hindlimb unloaded mice, the soleus muscle displays decreased capillary volume and capillary luminal diameter along with muscle atrophy [266]. Accordingly, the number of apoptotic endothelial cells and expression of anti-angiogenic factors, such as p53 and thrombospondin-1, were significantly increased, while VEGF production was dampened [266]. Oral gavage of Brazilian propolis (500 mg/kg, twice a day, for 2 weeks) restored the impairments induced by hindlimb unloading [266]. Thus, propolis has the potential to stimulate vascular regeneration. Additionally, we demonstrated that Brazilian green propolis ethanol extract (100 μg/mL) induced various chemokines, such as CCL-2 and CCL-5, in C2C12 myoblasts via NF-κB activation [237]. Furthermore, supernatant of propolis-conditioned C2C12 cells stimulated migration of RAW264 macrophages, and production of vascular endothelial cell growth factor (VEGF)-A, and matrix metalloprotease (MMP)-12, both of which contribute to angiogenesis [237]. Therefore, propolis might prompt stromal cells, including myoblasts, to regenerate capillaries in diabetic mice. In contrast, anti-angiogenic effects of propolis have also been reported. Izuta et al. demonstrated that Chinese red propolis ethanol extract (0.3–3.0 μg/mL) and CAPE (1–10 μM) impeded the VEGF-elicited in vitro tube formation of human umbilical vein endothelial cells (HUVECs) in a dose-dependent manner [267]. They also indicated that the Chinese propolis extract and CAPE inhibited VEGF-dependent proliferation of HUVECs [267]. Further detailed studies using animal models are required to clarify how to apply propolis to overcome capillary defects in diabetic conditions.

## 5. Diabetic Complications

Diabetes causes hyperglycemia, leading to excess production of ROS and AGEs. Both ROS and AGEs further accumulate in cells through positive feedback loops. Eventually, damaged cells are not able to maintain functional integrity, occasionally leading to cell death. Since ROS production and AGE–RAGE ligation are elevated in certain types of cells, such as endothelial cells, kidney cells, and retinal epithelial cells [268,269], cell defects in diabetic patients become apparent. Additionally, an overload of carbohydrates and lipids also causes insulin resistance, resulting in dysfunction of energy metabolism-competent organs such as the liver, skeletal muscle, and adipose tissues [270]. Consequently, dyslipidemia along with hyperglycemia causes defects in blood circulation, which is a primary symptom of several geriatric diseases, such as atherosclerosis, stroke, myocardial infarction, liver cancer, and Alzheimer’s disease [271,272]. In this context, the term “diabetic complications” covers a wide variety of diseases. In this section, more narrowly defined diabetic complications, namely diabetic nephropathy, retinopathy, wound healing delay, and NAFLD, are given particular focus. 

### 5.1. Diabetic Nephropathy

#### 5.1.1. Pathology and Models of Diabetic Nephropathy

Diabetic nephropathy is one of the common complications of diabetes mellitus. In many cases, histopathological approaches, as well as blood biochemical examinations, are used to evaluate disease state. Blood urea nitrogen (BUN), serum creatinine, and proteinuria levels are frequently monitored [48,130,131,146]. In addition to the high blood pressure generated by obesity, continuous high blood glucose severely damages kidney cells and consequently decreases glomeruli. Thus, clinical intervention to normalize blood glucose levels prevents the progression of diabetic nephropathy [273]. In the process of diabetic nephropathy, AGEs gradually accumulate in the kidneys, and eventually increase to ~2-fold normal levels in diabetic patients without end-stage renal disease [274]. The involvement AGEs in diabetic nephropathy has been observed using several animal models. Administration of AGEs led to progression of diabetic nephropathy in STZ-treated rats and mice [275,276]. In agreement with these data, transgenic overexpression of methylglyoxal-detoxifying enzyme glyoxalase-1 diminished early renal dysfunction markers, including elevation of urinary albumin, osteopontin, kidney-inflammation-molecule 1, and nephrin in T1DM rats [277]. In db/db mice, AGEs suppress podocyte autophagy, which is necessary for maintenance of normal kidney function [278]. The AGE-elicited autophagy of podocytes is mediated through hyperactivation of mTOR and subsequent inhibition of nuclear translocation of pro-autophagic transcription factor EB (TFEB) [278]. Considering the prerequisite roles of AGEs in diabetic nephropathy, modulatory agents for AGE production or RAGE activation are expected to be used as drugs for the disease. Thus, monitoring AGE or AGE-elicited signaling is useful to evaluate progression of nephropathy in experimental animal models.

So far, the effects of AGEs on kidney have been extensively investigated using isolated renal cells. AGEs stimulate mesangial cells to produce fibronectin, collagen IV, and TGF- β1, all of which are pivotal for diabetic kidney fibrosis [279,280,281]. Activation of protein kinase C (PKC), extracellular signal-regulated kinase (ERK), and sphingosine kinase 1 are postulated to participate in AGE-induced expression of fibronectin and TGF-β1 in mesangial cells [280,281,282]. Podocytes, which are determinants for proteinuria, are another major target cell type of AGEs. AGEs induce apoptosis via activation of transcription factor forkhead box protein O4 (FoxO4) [283] and inhibit migration and cell adhesion ability in a neuropillin-1-dependent manner [284]. Moreover, AGEs also promote epithelial-mesenchymal transformation (EMT) in podocytes by activation of NF-κB and zinc finger E-box-binding homeobox 2 (Zeb2), followed by an increase in glomerular permeability [285]. In diabetic conditions, epithelial cells in the proximal uriniferous tubule also undergo transdifferentiation into myofibroblasts, which participate in fibrosis in progressive chronic kidney disease [45,286]. Transdifferentiation of epithelial cells into myofibroblasts can be evaluated by assessing the expression profile of cellular markers: epithelial markers comprise E-cadherin, zonula occludens-1 (ZO-1), cytokeratin, connective tissue growth factor (CTGF), tissue inhibitor of metalloprotease-2 (TIMP-2) and collagen IV, while mesenchymal/myofibroblast markers comprise α-smooth muscle actin (α-SMA), fibroblast-specific protein-1 (FSP-1), N-cadherin, vimentin, Snail, and membrane type 1-matrix metalloprotease (MT1-MMP) [45,286].

#### 5.1.2. Effects of Propolis and Propolis-Derived Compounds on Diabetic Nephropathy

Several reports have demonstrated that propolis treatment improves the progression of nephropathy in diabetic models. Many articles describing the curative effects of propolis on diabetic nephropathy concomitantly show alterations in the activities of antioxidative enzymes and lipid peroxidation [130,131,138,156,162,258]. Therefore, oxidative stress is one of the crucial therapeutic targets of propolis in diabetic nephropathy. Zhu et al. compared the therapeutic effects of crude extracts of Chinese or Brazilian propolis (100 mg/kg i.g. for 8 weeks) on nephropathy in STZ-induced diabetic rats. Brazilian propolis significantly retarded the increases of BUN, serum creatinine, and urinary albumin-excretion rate (UAER), while Chinese propolis only modified the UAER [130]. Contrarily, the same group also showed that restoration of the STZ-induced histological changes was more prominent with Chinese propolis than with Brazilian propolis: Chinese propolis repressed the proliferation of mesangial cells and vacuolization of renal tubular epithelial cells, whereas Brazilian propolis only improved the response of mesangial cells [131]. Another report indicated that administration of Brazilian propolis extract (100–300 mg/kg/day for 40 days) restored kidney weight and BUN and serum creatinine levels [138]. Likewise, oral treatment with Saudi Arabian propolis decreased BUN, serum creatinine, serum uric acid, and urinary creatinine [135]. Accordingly, propolis treatment improved the histopathological images of the renal cortex of STZ-treated rats along with the increases in serum sodium and potassium ions. Moreover, Iranian propolis extract (100 or 200 mg/kg/day i.g. for 6 weeks) suppressed STZ-induced kidney atrophy, enlargement of glomerular area, and thickening of glomerular basement membrane [136]. 

Of the many chemicals in propolis, chrysin has been most studied for application in diabetic nephropathy. Chrysin (40 mg/kg/day i.g.) suppressed increases of BUN, serum creatinine, and proteinuria in HFD/STZ-induced T2DM mice, at least until 16 weeks [161]. Accordingly, chrysin treatment abated tubular atrophy, proliferation of mesangial cells, enlargement of glomeruli, and glomerular basement membrane thickening [161]. On the other hand, chrysin strongly attenuated local and systemic production of proinflammatory cytokines in the diabetic mice [161]. Chrysin also reduced production of TGF-β, fibronectin, and collagen IV in the kidneys of diabetic mice [161]. Given that TGF-β is a prerequisite cytokine for tubulointerstitial fibrosis, chrysin seems able to mitigate renal fibrosis in diabetic conditions. 

**Figure 5:**
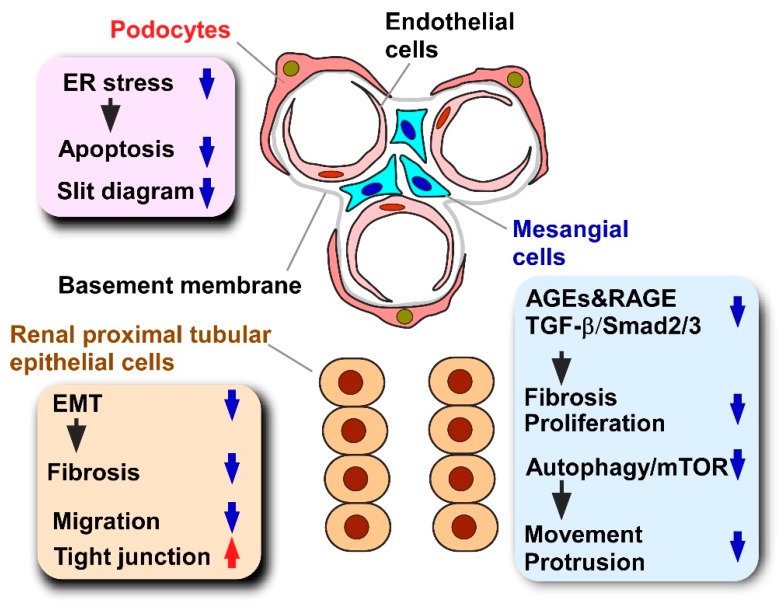
The effects of chrysin, a natural flavonoid in propolis, on diabetic nephropathy are depicted based on publications by Kang and colleagues [45,46,47]. Chrysin administration improved the diabetic nephropathy of *db/db* mice. Chrysin ameliorated dysfunction of podocytes, mesangial cells, and renal proximal tubular epithelial cells. Blue and red arrows represent changes by chrysin in diabetic situations, such as high glucose or high AGE conditions. AGEs, advanced glycation end products; EMT, epithelial-mesenchymal transition; ER, endoplasmic reticulum; RAGE, receptor for AGEs.

The detailed cellular and molecular effects of chrysin in renal cells were extensively investigated by Kang and colleagues (Figure 5) [45,46,47,48]. 

Oral administration of chrysin (10 mg/kg/day for 10 weeks) dramatically inhibited the increase of α-SMA and FSP-1 in the renal epithelial cells of *db/db* mice [45]. In addition, chrysin decreased TIMP-1 in the kidneys of *db/db* mice, while chrysin also augmented expression of MT1-MMP and the tight junction protein ZO-1 [45]. In addition, chrysin (1–20 μM) also disturbed the glucose-increased migration of the human renal proximal tubular epithelial cells along with repression of MMPs [45]. These data collectively suggest that chrysin attenuates the diabetic destruction of the solid renal tubular epithelial structure. To support this, chrysin modulated high glucose (33 mM) elicited expression changes in N-cadherin, FSP-1, collagen IV, and MMPs in the renal epithelial cells, indicating a suppressive role of chrysin on the EMT of epithelial cells [45]. On the other hand, chrysin also modified characteristics of mesangial cells. Chrysin (1–20 μM) reduced the glucose-induced production of AGEs and RAGE in human renal mesangial cells [46]. Moreover, chrysin also counteracted glucose- or AGE-induced expression of α-SMA, FSP-1, and collagen-I and –IV in the cultured mesangial cells, indicating that chrysin hampered the proliferation and extracellular matrix (ECM) production of mesangial cells [46]. The reduction of ECM from activated mesangial cells seems to be attributed to the blunting of TGF-β signaling. Indeed, chrysin markedly decreased TGF-β receptors in cultured mesangial cells, followed by insufficient translocation of Smad2/3 to the nucleus [46]. In addition to repression of fibrosis, chrysin also disturbed the AGE- or glucose-induced migration of renal epithelial cells and mesangial cells [45,47]. Meanwhile, chrysin diminished the hyperglycemia-related increase of autophagy-related proteins, such as beclin-1, LCI, and LCII, along with dephosphorylation of mTOR. Considering that autophagy is responsible for AGE-induced changes in the cytoskeleton of mesangial cells, chrysin modulates mesangial cell movement via the disruption of autophagy [47]. Additionally, podocytes are another possible target of chrysin. Chrysin (10 mg/kg/day p.o. for 10 weeks) reduced apoptotic podocytes as well as podocyte foot process effacement in *db/db* mice [48]. Chrysin also restored production of the slit diagram proteins nephrin and podocin [48]. In vitro experiments also provided evidence that chrysin ameliorated podocyte apoptosis and protein kinase R-like endoplasmic reticulum kinase (PERK)-dependent endoplasmic reticulum (ER) stress [48]. Taken together, these data indicate that chrysin exerts nephroprotective effects via maintenance of integrity of renal epithelial cells, mesangial cells, and podocytes.

CAPE (10 μmol/kg/day i.p. for 4 weeks) was also reported to have preventive effects on diabetic nephropathy evoked by high fat and sugar diet containing 0.1% cadmium chloride (Cd) [162]. CAPE administration completely abolished HFD/sugar/Cd diet-induced morphological changes in the renal tissues, such as renal tubular necrosis and glomerulus atrophy [162]. Accordingly, CAPE also dampened the increase of urinary albumin and renal MDA, and the decrease of kidney weight, nitric oxide content, and activities of renal antioxidant enzymes in the diabetic model [162]. Ultra-performance liquid chromatography coupled with quadrupole time-of-flight mass spectrometry (UPLC-Q-TOF-MS) analysis of the serum demonstrated that CAPE markedly reduced bioactive lipids, including arachidonic acids and 1-hexadecanoyl-sn-glycerol-3-phosphorylcholine [162]. Thus, CAPE might retard Cd-enhanced diabetic nephropathy via the metabolism of bioactive lipids and arachidonic acids

Pinocembrin, an antioxidant flavonoid, has also therapeutic effects on diabetic nephropathy. Oral administration of pinocembrin (10 mg/kg/day) immediately after STZ-treatment drastically inhibited the appearance of lobulation of the glomeruli, mesangial expansion, ECM accumulation, and glomerular basement membrane thickening in rats [146]. The curative effects of pinocembrin were apparent in the only preventive scheme because pinocembrin treatment failed to cure preexisting diabetic nephropathy [146]. Accordingly, pinocembrin also reduced the increases in estimated glomerular filtration rate, urinary volume, and urinary protein in the preventive scheme, although it worsened urinary volume and urinary protein in the corrective scheme [146]. Meanwhile, renal damage markers, namely kidney injury molecule-1 (Kim-1), neutrophil gelatinase-associated lipocalin (NGAL), and *N*-acetyl-β-d-glucosaminidase (NAG) in urine were also decreased only in the preventive scheme, while in the corrective scheme the increases worsened [146]. Thus, pinocembrin is useful for the prevention of diabetic kidney failure. 

### 5.2. Diabetic Retinopathy

#### 5.2.1. Pathology and Models of Diabetic Retinopathy

More than 60% of patients with type 2 diabetes will suffer from retinopathy after 20 years of having diabetes [287]. Since diabetic retinopathy progresses through the interaction of various retinal cells including endothelial cells, pericytes, and infiltrated immune cells, experimental approaches using animal models are useful to evaluate the effects of propolis on the disease. In diabetic conditions, mitochondria and Nox produce ROS in the retina [12]. In fact, oxidative stress was clearly observed in the retinas of STZ-induced diabetic Lewis rats [288]. ROS activates several biochemical pathways, including the AGE-RAGE pathway and the PKC pathway, which leads to further accumulation of intracellular ROS [12]. In addition to evoking the cell death of pericytes and endothelial cells [289,290], ROS enhances leukocyte adhesion to endothelial cells by increasing the expression of intracellular adhesion molecule (ICAM) and CD18 [12]. Consequently, infiltrated leukocytes cause low-grade inflammation, possibly by sensing apoptotic cell death [12]. Moreover, local production of proinflammatory cytokines, such as TNF-α- and IL-1β, evokes activation of NF-κB, leading to aggravation of inflammation [12]. Eventually, breakdown of the blood-retina-barrier (BRB) occurs through loss of tight junction proteins ZO-1 and occludin [12]. In rat models, the BRB can be analyzed by extravasation of Evans blue in the retina after tail vein injection [129]. Histologically, increased cell death of pericytes and endothelial cells has been observed in an alloxan-induced diabetic model [291]. Moreover, the prominent loss of capillary pericytes causes microaneurysm formation and retinal basement membrane thickening [292]. 

#### 5.2.2. Effects of Propolis and Propolis-Derived Compounds on Diabetic Retinopathy

Shi and colleagues investigated the effects of Chinese propolis ethanol extract (200 mg/kg/day p.o. for 12 weeks) on retinopathy in STZ-induced diabetic rats [129]. In parallel with the slight decrease of FBG and Hb1Ac and the nearly complete restoration of serum MDA, ROS, and RNS, propolis counteracted diabetes-induced photoreceptor cell death, resulting in prevention of retinal membrane thickening [129]. Accordingly, propolis treatment reduced BRB permeability in diabetic rats [129]. Additionally, propolis reinstated the expression of occludin and ZO-1 in the retina [129]. Therefore, Chinese propolis seems to protect against oxidative stress-induced BRB breakdown by ameliorating the dysfunction of the tight junctions of retinal cells. 

A recent report also indicated the therapeutic potential of chrysin in diabetic retinopathy [293]. High glucose concentration (33 mM) induced AGEs and RAGE in human retinal pigment epithelial (PRE) cells following the emergence of ER stress. High glucose disturbed the expression of visual cycle enzymes [such as retinoid isomerohydrolase (PRE65), lectin acyltransferase (LRAT), and retinol dehydrogenase 5 (RDH5)] and retinoid binding proteins [cellular retinoid binding protein (CRBP), cellular retinaldehyde-binding protein (CRALBP), and interphotoreceptor retinoid-binding protein (IRBP)] and vitamin A receptor (STRA6) in PRE cells [293]. Chrysin (1–20 μM) reversed the glucose-induced expression changes in visual cycle components [293]. Chrysin also recovered production of insulin-like growth factor 1 (IGF-1) and pigment epithelium-derived factor (PEDF) in PRE cells, although VEGF was downregulated [293]. In accordance with the cellular model, an oral intubation feeding of chrysin (10 mg/kg for 10 weeks) dampened the impaired expression of visual cycle components and rhodopsin in the eyes of *db/db* mice, along with elimination of AGE accumulation and ER stress [293]. Moreover, chrysin administration also completely inhibited changes in the outer layer thickness in *db/db* mice [293].

The protection of PRE cells from oxidative stress by other flavonoids in propolis was also assessed. Similar to anthocyanin, resveratrol, and lutein, kaempferol (20 or 50 nM) protected human PRE cell line APRE-19 from H_2_O_2_-induced death [294]. Concomitantly, kaempferol attenuated H_2_O_2_-induced expressional changes in Bcl2, Bcl-2-associated X protein (Bax), and caspase in APRE-19 cells, followed by decrement of annexin V^+^, propidium iodide^+^ apoptotic cells [294]. Additionally, pretreatment with kaempferol (50 μL of 0.3% solution, i.v.) prevented sodium iodide-induced apoptosis of retinal pigment epithelial cells, as well as morphological changes in the retinal epithelia in rats [294]. Considering that oxidative stress is evident in diabetic patients, kaempferol might be applicable for diabetic retinopathy through reduction of oxidative stress in retinal epithelial cells.

### 5.3. Delayed Wound Healing

#### 5.3.1. Pathology and Models of Diabetes-Deteriorated Wound Healing

In a similar context to other diabetic complications, hyperglycemia causes damage to endothelial cells in the skin and mucosa. Since production of VEGF in endothelial cells and fibroblasts is downregulated in diabetic patients/animals, remodeling of the microvasculature is severely interrupted in wounds [244]. Moreover, diabetic individuals display aberrant production of ECM in fibrocytes through interference with signals of growth factors such as TGF-β, fibroblast growth factor (FGF), and hepatocyte growth factor (HGF) [244,295,296]. On the contrary, hypoxia stimulates fibroblasts to produce MMPs, resulting in digestion of ECM [244]. In addition, neutrophils infiltrate the lesion excessively and contribute to impaired wound healing [297]. As a result, diabetic individuals are vulnerable to injury of the skin and mucosa, and sometimes suffer from ulceration [298]. To examine the wound healing effects of propolis in vivo, propolis-containing hydropropyl methylcellulose gel [148,149] or propolis extracts [150] are used to topically treat artificial lesions in STZ-induced diabetic animals. In these studies, the wound healing rate is calculated and pathohistological analysis is conducted [148,149,150]. In addition, the local content of MMPs, ECM, cytokines, and growth factors are measured [148,149,150,299]. Slow wound healing in diabetic patients is occasionally accompanied with severe bacterial infections [244]. To this point, propolis might be an ideal ointment material because it is a natural product with low toxicity and bactericidal effects.

#### 5.3.2. Effects of Propolis and Propolis-Derived Compounds on Wound Healing

**Figure 6:**
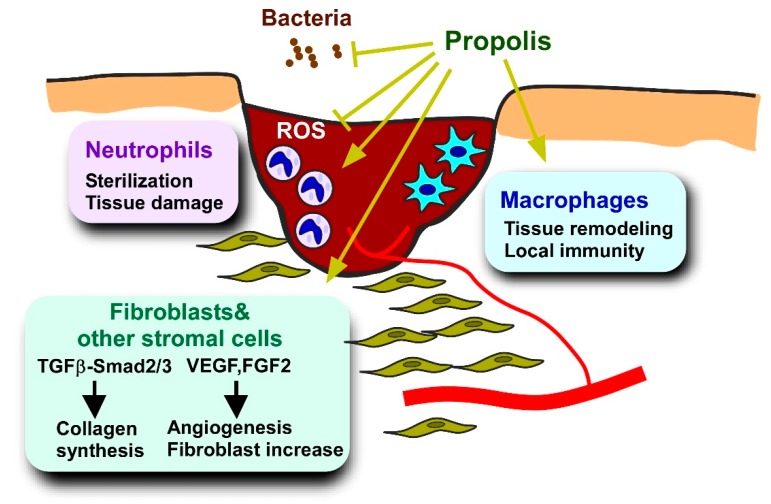
Effects of propolis on diabetes-worsened wound healing. Propolis mitigates excess activation of neutrophils to avoid tissue damage. Additionally, propolis stimulates macrophage infiltration into the lesion to remodel the tissues. Propolis also activates TGF-β signaling in fibroblasts, resulting in potentiation of collagen synthesis. Moreover, accumulation of VEGF and FGF2 might contribute to angiogenesis. Since propolis is a natural antimicrobial agent, it may help to protect against microbial infection. Collectively, propolis has potential to accelerate wound healing in diabetic animal models.

In diabetic patients, defects in local circulation retard wound healing [296]. In severe cases, ulcers are observed on the foot. Accumulating evidence indicates that topical treatment of propolis augments wound healing (Figure 6). 

For instance, McLennan et al. reported that topical application of propolis (“Honey Spring” product; 20 μL/wound) ameliorated the delayed re-epithelization and epithelial closure of a scratched lesion on STZ-induced diabetic rats [151]. In the lesions, propolis stimulated macrophage infiltration, while blunting the accumulation of neutrophils to avoid tissue damage by ROS [151]. Indeed, propolis diminished the increase of myeloperoxidase (MPO) and also generated an increase of proliferative cells in the lesions [151]. Thus, this study implied that propolis prevents attacks by neutrophils and promotes tissue repair by macrophages. In a similar context, “honey spring” propolis decreased proinflammatory cytokines, such as IL-1β, IL-6, and TNF-α, and also attenuated a cutaneous proteolytic enzyme, MMP9 [150]. Similar data have been reported using a bacterial cellulose membrane with 1% adsorbed Brazilian red propolis [152]. In the study, STZ-treated rats showed better closure of the lesions after treatment with the bacterial membrane with absorbed propolis than either ethanol or butanol extract [152]. The butanol extract-containing membrane markedly suppressed edema, hyperemia, and exudation, while the ethanol extract-containing membrane showed reduced but significant effects [152]. Furthermore, both extract-containing membranes also stimulated wound contraction accompanied with an enhanced induction of TGF-β [152]. On the other hand, the propolis-containing membranes increased MPO activity and TNF-α levels in the lesions [152]. Thus, adequate accumulation of neutrophils in the lesions might participate in rapid wound healing in this model. Other reports focused more on the capacity of propolis to stimulate production of ECM. “Honey spring” propolis reversed aberrant expression of TGF-β and subsequent Smad2/3 phosphorylation in STZ-induced diabetic mice [150]. Accordingly, propolis restored insufficient type I collagen production in the wound while it inhibited the detrimental increase of MMP9 [150]. Propolis extracts have also been applied to an oral traumatic ulcer model. Indonesian propolis-absorbed hydroxypropyl methylcellulose gel was applied to artificial ulcers made on the lower lip of STZ-treated Wister rats [148,149]. After three days of treatment, FGF-2 and VEGF were significantly induced in the lesions of the propolis-treated group compared with the control group, and the changes were sustained for nine days after treatment [148,149]. In accordance with these findings, the number of fibroblasts was more profoundly increased in the propolis-treated group [149]. In addition to biological responses in diabetic patients, propolis also has preventive effects on local bacterial infections. The application of poly(vinyl alcohol)-blended cellulose membranes with absorbed Brazilian propolis and vitamin C improved healing of back injuries compared with control membranes in STZ-induced diabetic mice [153]. In this case, the propolis-containing membrane decreased the number of bacteria identified by a swab test [153]. In conclusion, topical treatment of propolis accelerates wound healing through its immunomodulatory, fibroplastic, and microbicidal activities.

### 5.4. NAFLD and Hepatic Steatosis

#### 5.4.1. Animal and Cellular Models

NAFLD is one of the most common chronic liver disorders. In the early phase, the liver exhibits only simple steatosis, which subsequently develops into steatohepatitis and fibrosis. In the case of severe non-alcoholic steatohepatitis, hepatitis progresses to cirrhosis, occasionally leading to hepatic cancer. More than 70% of type 2 diabetes patients whose Hb1Ac level is ≥8.5% display symptoms of NAFLD [300]. Therefore, NAFLD model animals have usually been generated simply by feeding high-carbohydrate or HFD [17]. Since C57BL/6 mice are more sensitive to the obesogenic diet, C57BL/6 mice have been used for studies of NAFLD and non-alcoholic steatohepatitis (NASH), a progressive inflammatory type of NAFLD [301]. Monogenic mouse models, such as ob/ob and db/db mice, have been also employed [17]. As with NAFLD patients, the golden standard of diagnosis for NAFLD in mice is histological assessment to monitor steatosis. Alternatively, blood biochemical indices such as triglyceride, LDL-C, HDL-C, alanine amino transferase (ALT), and aspartate amino transferase (AST) may be used [109,155,156,159,165,262,302,303]. Recently, an emerging body of evidence indicates that several microRNAs (miRs), including miR-122, are relatively rapid and sensitive blood parameters of NAFLD in rats [304]. Additionally, expression of several catalytic enzymes or transcription factors for gluconeogenesis and lipid metabolism have also been measured to evaluate the effects of propolis and derived compounds on NAFLD or hepatic steatosis [108,156]. 

#### 5.4.2. Effects of Propolis and Propolis-Derived Compounds on NAFLD

Kismet et al. focused on the effects of Turkish propolis (100 or 200 mg/kg/day for 2 weeks) on NAFLD using a rat model given fatty diet, sucrose-containing drinking water, and corn kernels [303]. In this model, propolis treatment initiated when fatty liver formations became apparent. Propolis treatment significantly lowered serum triglyceride, total cholesterol, and non-HDL-C in a dose-dependent manner [303]. Moreover, propolis also markedly decreased serum ALT, ALP, IL-6, and TNF-α levels, indicating that propolis reduced NAFLD-induced hepatic damage [303]. Concomitantly, propolis mitigated steatosis, ballooning, and lobular inflammation in the NAFLD model rats [303]. Therefore, Turkish propolis inhibited progression of NAFLD in the rat models. On the other hand, Brazilian propolis (200 mg/kg p.o. for 5 weeks) attenuated *P. gingivalis* elicited hepatic steatosis, along with diabetes-like aberrant expression of hepatic metabolism-associated genes [262]. Likewise, Brazilian propolis extract also prevented hepatic steatosis of HFD-fed mice. Gavage of 5 or 50 mg/kg of Brazilian propolis for 10 days decreased liver weight and hepatic triglyceride content by more than 17% and 18%, respectively, in HFD-fed mice [108]. In this setting, propolis reduced hepatic expression of sterol regulatory element binding protein (SREBP)-1 and SREBP-2, both of which are vital for fatty acid synthesis and cholesterol synthesis, respectively [108]. Conversely, the propolis extracts also downregulated fatty acid synthesizing enzymes such as acetyl-CoA carboxylase α and fatty acid synthase (FAS) in the liver [108]. In addition, propolis also tended to control gene expression of cholesterol metabolism-associated enzymes, such as 3-hydroxy-3-methylgultanyl coenzyme A reductase and squalene epoxidase [108]. Therapeutic effects of Croatian propolis on diabetes-induced hepatic defects were also investigated using alloxan-induced diabetic mice [156]. Water and ethanol extracts of Croatian propolis (50 mg/kg/day i.p. for 7 days) partially inhibited serum AST and ALT in the mice. Accordingly, propolis protected vacuolization of hepatocytes in the diabetic mice [156]. Since propolis decreased MDA content in the liver of the diabetic mice, the authors assumed that the removal of local oxidative stress accounts for the hepatoprotective ability of propolis [156].

Regarding propolis-derived compounds, chrysin has been shown to have anti-NAFLD activity in a rat model [302]. Oral administration of chrysin (25–100 mg/kg/day for 8 weeks) improved fasting glucose, insulin, HOMA-IR, total cholesterol, HDL-C, and non-HDL-C levels of fructose (for 16 weeks) and HFD (8 weeks)-induced NAFLD rats [302]. In these rats, restoration was apparent in the liver index (which is calculated as the percentage of liver weight to body weight), liver enzymes (ALT, AST, alkaline phosphatase, and γ glutamyl transferase), triglyceride, FFAs, and cholesterol in the liver [303]. Since chrysin attenuated accumulation of MDA, AGEs, and proinflammatory cytokines in the liver in a dose-dependent manner, propolis seems to ameliorate oxidative stress-induced hepatic inflammation [302]. Moreover, chrysin also decreased SREBP-1c levels in the diabetic liver, while it increased the level of PPARα [302]. By consequence, propolis remarkably reversed steatosis, ballooning, and lobular inflammation in the liver along with decreasing liver collagen [302]. Therefore, chrysin improves NAFLD through antioxidation, anti-glycation, anti-inflammation, and anti-steatosis.

## 6. Effects of Propolis on Diabetes and Diabetic Complications in Humans

As previously mentioned (see Section 2.1), there are considerable biological differences between animal models and human patients. Thus, one should consider the limitations of using animal models to study the therapeutic effects of propolis extracts and propolis-derived compounds for use in human patients. Prior to administering propolis to patients, sufficient clinical trials of propolis extracts and propolis-derived compounds will eventually be required. In this section, I have provided an overview of human clinical data investigating the effects of propolis on diabetes and diabetic complications.

### 6.1. Blood and Urinary Indices

So far, numerous reports have demonstrated that propolis supplementation improves several blood indices in T2DM patients (Table 6). For instance, a double-blind, placebo-controlled clinical trial conducted over 90 days indicated that Iranian propolis (1 g/day, *n* = 50) significantly improved levels of HbA1c, blood insulin, HOMA-IR, homeostatic model assessment-β(HOMA-β), and 2-h postprandial (2 hp) in comparison with the placebo group (*n* = 44) [305]. The propolis treatment also increased blood HDL-C, whereas it did not significantly impact body weight, BMI, blood triglyceride level, total cholesterol, LDL-C, or VLDL-C [306]. In this condition, blood C-reactive protein (CRP) and TNF-α levels were decreased by 30% and 16% following propolis treatment, respectively [305]. Therefore, Iranian propolis treatment mitigated systemic inflammation in T2DM. Similarly, administration of a propolis-containing pill (propolis 900 mg/day for 12 weeks, *n* = 30) produced by an Iranian company was found to remarkably decrease FBG, HbA1c, blood total cholesterol, and LDL-C, while it failed to affect blood triglyceride level, HDL-C, VLDL-C, and insulin resistance indices in T2DM patients [306]. Moreover, another report demonstrated that capsules containing Iranian propolis (1.5 g/day for 8 weeks, *n* = 30) led to decreased FBG, blood insulin, HbA1C, and insulin resistance compared with the placebo treated group (*n* = 30) [307]. Despite some differences in the changes in lipid parameters and insulin indices between these three reports, Iranian propolis has been shown to have beneficial glycemic effects in T2DM patients. The administration of Iranian propolis increased the total antioxidant activity, SOD, GPx, and CAT in the patients along with reductions of oxidized LDL and fructosamine [307,308]. These results imply that the antidiabetic activity of Iranian propolis might be ascribed to its antioxidant activity. Based on simple comparison of the data from previous studies, the therapeutic effects of Chinese propolis in T2DM patients are likely to be milder than those of Iranian propolis [305,306,307,308,309]. Chinese propolis supplementation (900 mg/day for 18 weeks, *n* = 25) had negligible effects on the levels of serum glucose, glycosylated hemoglobin, insulin, FRAP, MDA, SOD, GPx, IL-1β, and TNF-α in T2DM patients compared with the control group (*n* = 30) [309]. On the other hand, serum GSH was elevated from 2.2 g/L to 7.4 g/L, accompanied by a reduction of lactate dehydrogenase (LDH) [309]. Since serum polyphenols and flavonoids were increased after treatment with Chinese propolis, supplementation of Chinese propolis seems to inhibit tissue damage in T2DM patients through the accumulation of circulating botanical antioxidants. A modest influence on blood indices has also been reported based on clinical trials using Brazilian green propolis [310,311]. Administration of Brazilian propolis extract in T2DM patients (226.8 mg/day for 8 weeks, *n* = 41) did not result in significant improvements in HOMA-IR, FBG, HbA1c, blood insulin, total cholesterol, HDL-C, LDL-C, triglyceride, remnant-like particle cholesterol, TNF-α, IL-6, CRP and urinary albumin excretion compared to the placebo group (*n* = 39) [310]. Additionally, T2DM patients receiving Brazilian green propolis (900 mg/day, *n* = 32) or placebo (*n* = 33) did not demonstrate distinguishable differences in glucose metabolism indices such as blood glucose, glycosylated hemoglobin, insulin, aldose reductase, or adiponectin [311]. On the other hand, treatment with Brazilian green propolis for 18 weeks significantly increased GSH and total polyphenol in the blood concurrently with decreases in circulating carbonyls, LDH, and TNF-α [311]. Therefore, like Chinese propolis, Brazilian green propolis seems to exhibit mild anti-diabetic effects through antioxidant activity [309,311]. Additionally, commercially available propolis (“BioPropolis”) was also administered in a clinical trial for diabetic patients [312]. In this study, capsules containing BioPropolis (400 mg/day, *n* = 26) or placebo (*n* = 24) were administered to T2DM patients suffering from periodontitis for 3 or 6 months [312]. Treatment with propolis lowered the levels of fasting plasma glucose and HbA1c at the both time points, while the placebo did not [312].

Blood indices have been used to evaluate the effects of propolis on tissue damage by T2DM. Administration of Iranian propolis (1 g/day for 90 days) clearly decreased the circulating levels of BUN, AST, and ALT in T2DM patients, indicating that Iranian propolis has hepatoprotective and renoprotective effects [305]. Accordingly, the estimated glomerular filtration rate (eGFR) was sustained during propolis treatment while eGFR was significantly decreased in placebo-treated patients [305]. Likewise, Brazilian propolis (226.8 mg/day for 8 weeks) sustained blood uric acid levels while these were increased in the placebo group during the trial period [310]. Accordingly, Brazilian propolis interrupted the decrement of eGFR in T2DM patients [310]. Moreover, another report has suggested curative effects of propolis (500 mg/day) on proteinuria, aberrant eGFR, and urinary albumin-to-creatinine ratio in chronic kidney disease patients, including diabetic patients [313]. Additionally, “BioPopolis” (400 mg/day) improved periodontal parameters, such as probing pocket depth and clinical attachment, in agreement with reduction of blood N6-(carboxymethyl)-l-lysine, a predominant AGE [312].

### 6.2. Wound Healing

The effects of topical treatment of foot ulcers of T2DM patients with propolis have been also examined. Topical application of “Honey spring” propolis to the foot ulcers of 24 T2DM patients for 6 weeks decreased ulcer area by 41% compared with the control group (*n* = 84) [314]. The numbers of patients whose ulcers had healed were significantly higher in the propolis-treated group than the control group at 4, 5, and 7 weeks after beginning the treatment, implying that propolis promoted wound healing [314]. In this trial, propolis was shown to reduce the bacterial load in the lesion [314]. Additionally, propolis also decreased the accumulation of active MMP-9 in post-debridement wound fluid [314]. Therefore, propolis improves wound healing through reduction of bacterial load as well as MMP-9-accelerated inflammation. In accordance with these results, topical treatment with 5% propolis water extract-containing ointment reduced ulcer size 1 week after initiation propolis treatment (*n* = 20) compared with control ointment treatment (*n* = 20) [315]. The numbers of patients showing ulcer discharge and erythema were diminished in the propolis-treated group (discharge, 5%; erythema, 0%) compared to the control group (discharge, 15%; erythema, 15%) [315]. More recently, the effects of Chilean propolis spray on diabetic foot ulcers have also been reported [316]. Chilean propolis spray reduced the average ulcer size and promoted fibrosis [316]. On the other hand, patients who received propolis spray exhibited increased tissue GSH as well as an increased GSH/glutathione disulfide ratio, indicating that propolis increased local antioxidant activity [316]. In the ulcer region, propolis treatment decreased TNF-α content as well as inducing a net increase in IL-10 content, suggesting the anti-inflammatory potential of propolis [316]. Collectively, topical treatment with propolis promotes wound healing of foot ulcers in T2DM patients. 

## 7. Perspectives

The U.S. National Library of Medicine governs a database named ClinicalTrials.gov, which is a repository of privately and publicly funded clinical studies [317]. As of November 2019, 46 human clinical trials using propolis or beeswax have been deposited. Among them, 21 studies have been completed. In the 21 completed studies, three studies were associated with T2DM: two focused on assessing the efficacy of propolis in the treatment of diabetic foot ulcers and one focused on glycemic control in patients suffering from T2DM and chronic periodontitis. Although the data from one of the completed studies has not yet been published in an article, the data from the other studies are available [312,316]. In the future, researchers will be able to easily and efficiently browse clinical data on propolis treatment deposited in the database. Although these clinical studies suggest potential applications of propolis in anti-diabetes therapies, they have not provided detailed clarification of the mechanisms underlying the curative effects of propolis. In this context, studies using animal models and cultured cells will be increasingly required as human trials grow in number. 

Emerging evidence suggests propolis to be a multi-potent medication for metabolic disorders. However, it is necessary to pay attention to the production area, because the constituents of propolis are determined by geographic factors, like *terroir* for wine. Moreover, the quality of propolis is affected by seasonal effects as temperature and precipitation strongly influence the condition of bees and plants. To circumvent the emergence of differences in therapeutic effects using different lots of propolis, standardization techniques should be established to monitor active ingredients of propolis, especially for medical purposes. Firstly, it will be necessary to establish standardization techniques for each propolis type produced in different geographic origins. For each type of propolis, it will be necessary to define “marker compounds” for quality control. Previously, Bankova and colleagues established rapid and low-cost spectrometric methods to quantify three main “marker” groups of poplar-type propolis, namely total flavone and flavonol groups, total flavanone and dihydroflavonols, and total phenolics [318,319]. They consider that measuring concentrations of the active compound groups, rather than those of individual components, is more realistic and more likely to be informative in the case of propolis validation [318]. On the other hand, highly sensitive detection of substances, including minor compounds, has also been employed for validation of propolis. In addition to gas chromatography-mass spectrometry [320,321], high resolution HPLC-coupled mass spectrometry and/or HPLC-coupled diode array detectors have recently been used to profile chemical compounds in propolis. For example, a liquid chromatography-diode array detector-quadrupole-time of flight (LC-DAD-QTOF) system has been employed to investigate the phytochemical composition of propolis from Northwestern Argentina [320]. In this study, the instrument detected several allergens derived from minor botanical sources of the propolis [322]. In addition to the chemical analytical techniques, evaluation of biological activities should be involved in the validation of propolis, especially for biomedical purposes. To evaluate this, antibacterial activity against *Escherichia coli*, *Staphylococcus aureus*, and *Candida albicans* has been analyzed for quality control of propolis [318,321]. Moreover, antioxidant activity of propolis or propolis-derived compounds has also been measured, frequently via colorimetric analysis using 2,2-diphenyl-1-picrylhydrazyl [110,133,146,156,321]. Given that the biological activity of propolis is not restricted to antibacterial and anti-oxidative effects, different approaches might be developed in the future. For example, activation of caspases, active status of insulin signaling, or cytokine production in certain cell lines might be applicable to the quality control of propolis.

Although pure chemicals isolated from propolis are better to use for especially medical purposes in the future, crude propolis extracts show more intense biological effects in several indices [113,164], and the exploration of novel bioactive substances in propolis is desirable. Additionally, it will be necessary to investigate the combinatory effects of propolis-derived compounds. A large proportion of previous articles have assumed that the effects of propolis on metabolic disorder are accounted for by antioxidant activity of flavonoids and natural phenols [129,130,131,132,134,137,138,139,146,156,159,162,163,202,258,259,260]. However, propolis-containing materials, such as APC and drupanin, function as ligands for nuclear receptors such as PPARγ and retinoid X receptor [50,118]. Moreover, recent metabolomics studies have shown that CAPE acts as a lipoxygenase inhibitor and Nrf2 activator [162]. Based on the fact that each propolis derivative has diverse molecular targets, synergism of compounds can be expected.

The major therapeutic effects of propolis are believed to be attributable to flavonoids and natural phenols, and both of these materials are believed to have a low toxicity and a low probability of side effects. However, we cannot exclude the possibility that propolis will have adverse effects on patients. Indeed, a human study has proven that propolis can be a skin allergen [323]. To avoid the harmful effects of propolis, comprehensive monitoring of biological effects seems to be important. Unfortunately, only a limited number of studies about the anti-diabetic effects of propolis employed -omics approaches, such as transcriptomics, proteomics, and metabolomics [109,162]. In accordance with the depreciation of the costs of these approaches, one can more freely gain access to information on the adverse effects of propolis in experimental models. On the other hand, -omics data might also help uncover unexpected beneficial effects of propolis in diabetic models. Additionally, -omics approaches may enable us to reduce the number of experimental animals, since they can provide large, objective data sets using a limited number of animals at the screening process. 

Although most previous papers have used vehicle controls, stress assessment might enforce the effectiveness of propolis and its derived chemical compounds, especially for in vivo studies. Since stress critically affects blood insulin level as well as glucose metabolism, accurate evaluation of the effects of propolis should be performed under the same stress situations between experimental groups. Propolis extracts usually have an intense odor and pungent taste. The pungent taste accounts for APC ligation to transient receptor potential channel ankyrin 1 (TRPA1) [324], which are abundant in the stomach and involved in stress-induced gastric mucosal lesions [325]. Hence, one should make efforts to exclude the “side effects” of propolis by using more adequate controls. Additionally, nonspecific inflammation should be also monitored during experimental series, since repeated intragastric or intraperitoneal injection of propolis per se might provide more opportunities for local tissue damage. By complete exclusion of artificial factors, more precise effects of propolis and its derived compounds can be ascertained. 

## Figures and Tables

**Table 1:** Effects of propolis or CAPE on obesity and dyslipidemia.

Administrated Materials	Mouse/Rat Models	Attenuation
Brazilian propolis	HFD	Body weight gain [108] Visceral adipose tissue weight [107,108] Serum triglyceride [107] Serum cholesterol [107]
Brazilian propolis	Leptin deficient	Visceral adipose tissue weight [109] Total cholesterol [109]
Croatian propolis	HFD	Body weight gain [110] Serum triglyceride [110] Serum LDL-C [110]
CAPE	HFD	Body weight gain [111] Visceral adipose tissue weight [111]

CAPE, caffeic acid phenethyl ester; HFD, high fat diet; LDL-C, low density lipoprotein-cholesterol; NEFAs, non-esterified fatty acids.

**Table 2:** Effects of propolis or propolis-derived compounds on adipocytokine expression.

Administrated Material	Model	Adipocytokine	Expressional Change
Brazilian propolis	3T3-L1 HFD mouse	Leptin	Increase [113]
CAPE	3T3-L1, ASCs-derived adipocytes	Leptin	Decrease [114,116]
Brazilian propolis	3T3-L1	Adiponectin	Increase [117]
Artepillin C	3T3-L1	Adiponectin	Increase [49,118]
Compound **1**	BM-derived adipocytes	Adiponectin	Increase [51]
CAPE	ASCs-derived adipocytes	Adiponectin	Increase [116]
CAPE	3T3-L1, ASCs-derived adipocytes	TNF-α	Decrease [114,116]
CAPE	3T3-L1	Resistin	Decrease [114]
CAPE	ASCs-derived adipocytes	IL-1β, IL-6, IL-8	Decrease [116]

CAPE, caffeic acid phenethyl ester; HFD, high fat diet; ASCs, adipocyte stem cells; BM, bone marrow.

**Table 3:** Experimental animal models used for assessments of propolis on diabetes.

Model	Primary Defect	Diabetes Type	References
Streptozotocin injection	β-cell destruction	Type 1	[129,130,131,132,133,134,135,136,137,138,139,140,141,142,143,144,145,146,147,148,149,150,151,152,153]
Alloxan injection	β-cell destruction	Type 1	[137,147,154,155,156]
S961 injection	β-cell destruction	Type 1	[128]
High fat diet with low dose streptozotocin injection	β-cell impairment Adipocity	Type 2	[157,158,159,160,161]
High fat diet	Adipocity	Type 2	[107,162,163]
*ob/ob* mouse	Aberrant leptin signal	Type 2	[109,164]
*db/db* mouse	Aberrant leptin signal	Type 2	[45,46,47,48]
OLETF rat	Aberrant cholecystokinin signal	Type 2	[165,166]

**Table 4:** Effects of propolis on oxidative stress indices of animal models.

Propolis Production Area	Diabetes Type	Tissues	Index	Change
Croatia	T1DM	Liver,	MDA	Decrease [156,258]
		Kidney		
		Spleen		
China	T1DM	Blood	MDA	Decrease [129,130,202]
			SOD	Increase [131,202]
			ROS, NOS, RNS	Decrease [129,202]
		Kidney	MDA	Decrease [131]
			CAT	Increase [131]
			GPx	Decrease [130]
		Liver	GPx	Increase [131]
Brazil	T1DM	Blood	MDA	Decrease [131,138]
			SOD, CAT, GSH	Increase [130,131,137]
			NOS	Decrease [137]
		Kidney	MDA	Decrease [130,131,138]
			SOD, CAT, GSH	Increase [138,195]
			GPx	Decrease [131]
		Liver	MDA	Decrease [131]
			SOD, GSH, GPx	Increase [130,131]
		Pancreas	MDA	Decrease [137]
			SOD	Increase [137]
Mexico	T1DM	Pancreas	SOD, CAT, GPx	Increase [134]
Malaysia	T1DM	Liver	MDA	Decrease [132]
			SOD, CAT, GSH, GPx, GST, GR	Increase [132]
			Total antioxidant activity	Increase [132]
		Pancreas	MDA	Decrease [133]
			SOD, CAT, GSH, GPx, GST, GR	Increase [133]
			Total antioxidant activity	Increase [133]
Iran	T1DM	Kidney	MDA	Decrease [136]
			SOD, GPx	Increase [136]
			FRAP	Decrease [136]
Nigeria	T1DM	Blood	MDA	Decrease [155]
			SOD	Increase [155]
Saudi Arabia	T1DM	Blood	MDA	Decrease [142]
		Blood	ROS	Decrease [142]
		Liver		
		Spleen		
		Bone marrow		
Saudi Arabia(?) ^1^	T1DM	Blood	MDA	Decrease [135]
			SOD, CAT, GST	Increase [135]
		Kidney	MDA	Decrease [135]
			SOD, CAT, GST	Increase [135]
Taiwan	T2DM	Blood	MDA	Decrease [159]
			SOD, GPx	Increase [159]

Alterations compared to the diabetic control are shown in the “Change” column. CAT, catalase; FRAP, ferric-reducing ability of plasma; GPx, glutathione peroxidase; GR, glutathione reductase; GSH, glutathione; GST, glutathione S-transferase; MDA, malondialdehyde; NOS, nitric oxide synthase; RNS, reactive nitrogen species; ROS, reactive oxygen species; SOD, superoxide dismutase; T1DM, type 1 diabetes mellitus; T2DM, type 2 diabetes mellitus. ^1^ Although the production area was unclear, the propolis was obtained in a shop in Saudi Arabia.

**Table 5:** Effects of propolis-derived compounds on oxidative stress indices of animal models.

Administrated Material	Diabetes Type	Tissues/Samples	Index	Change
Chrysin	T2DM	Skeletal muscle	MDA	Decrease [241]
			ROS	Decrease [241]
		Kidney	MDA	Decrease [242]
			SOD, CAT, GSH, GPx, GR	Increase [242]
Pinocembrin	T1DM	Blood	MDA	Decrease [246]
		Kidney		
Quercetin	T1DM	Blood	MDA	Decrease [245]
			RNS	Decrease [245]
		Pancreas	MDA	Decrease [245]
			SOD, CAT, GPx	Increase [245]
CAPE	T2DM and cadmium	Kidney	MDA, protein carbonyl	Decrease [171]
			SOD, CAT, GSH	Increase [171]
			RNS	Decrease [171]
	T1DM	Heart	MDA	Decrease [178]
			SOD, CAT	Decrease [178]
			GPx	Increase [178]
		Blood	LOOH	Decrease [252]
			RSH	Increase [252]
			ADMA	Decrease [252]
			RNS	Decrease [252]
		Pancreas	LOOH	Decrease [252]
			RSH	Increase [252]
			ADMA	Decrease [252]
			RNS	Decrease [252]
			NOS	Decrease [252]
			DDAH, HO-1, GGCL	Increase [252]

Alterations compared to the diabetic control are shown in the “Change” column. ADMA, asymmetric NG, NG-dimethyl-L-arginine; DDAH, dimethylarginine dimethylaminohydrolase; CAT, catalase; GPx, glutathione peroxidase; GGCL, γ-glutamyl-cysteine ligase; GR, glutathione reductase; GSH, glutathione; GST, glutathione S-transferase; MDA, malondialdehyde; HO-1, heme oxygenase-1; NOS, nitric oxide synthase; RNS, reactive nitrogen species; ROS, reactive oxygen species; SOD, superoxide dismutase; T1DM, type 1 diabetes mellitus; T2DM, type 2 diabetes mellitus.

**Table 6:** Effects of propolis on blood indices of type 2 diabetic patients.

Administrated Materials	Dose (Duration)	Number of Patients	Index	vs. before Treatment (Propolis Group)	vs. Placebo (after Treatment)	References
Iranian propolis	1 g/day (90 days)	Propolis: *n* = 50 Placebo: *n* = 44	HbA1c Insulin, HOMA-IR, HOMA-β, TNF-α	Decrease	Decrease	[305]
			BUN, ALT, AST, IL-1β	Decrease	Unchanged	[305]
			2hp, ALP, CRP	Unchanged	Decrease	[305]
			HDL-C	Increase	Increase	[305]
			Body weight, BMI, FBG, Triglyceride, Total-C, LDL-C, VLDL-C, Creatinine, Uric acid, IL-6	Unchanged	Unchanged	[305]
	900 mg/day (12 weeks)	Propolis: *n* = 30 Placebo: *n* = 27	FBG, HbA1c	Decrease	Decrease	[306]
			BMI	Decrease	Unchanged	[306]
			Total-C, LDL-C	Unchanged	Decrease	[306]
			Body weight, Triglyceride, HDL-C, VLDL-C	Unchanged	Unchanged	[306]
			Insulin	Increase	Unchanged	[306]
	1.5 g/day (8 weeks)	Propolis: *n* = 30 Placebo: *n* = 30	FBG, 2hp, Insulin, HOMA-IR, HbA1c	Decrease	Decrease	[307]
			TAC, SOD, GPx	Increase	Increase	[307]
	1.5 g/day (8 weeks)	Propolis: *n* = 30 Placebo: *n* = 30	Fructosamine Ox-LDL	Decrease	Decrease	[308]
			CAT	Increase	Increase	[308]
			Body weight, BMI	Unchanged	Unchanged	[308]
Chinese propolis	900 mg/day	Propolis: *n* = 25 Placebo: *n* = 30	GSH, Flavonoids, IL-6	N.S.	Increase	[309]
			LDH	N.S.	Decrease	[309]
			Blood glucose, Glycosylated hemoglobin, Insulin	Unchanged	Unchanged	[309]
			FRAP, Polyphenols, SOD, GPx, MDA, Carbonyls, IL-1β, TNF-α	N.S.	Unchanged	[309]
Brazilian green propolis	226.8 mg/day (8 weeks)	Propolis: *n* = 41 Placebo: *n* = 39	HOMA-IR, FBG, HbA1c, Insulin, Uric acid, Total-C, LDL-C, RLP-C, Triglyceride, TNF-α, IL-6, CRP	Unchanged	Unchanged	[310]
	900 mg/day (18 weeks)	Propolis: *n* = 33 Placebo: *n* = 32	GSH, Polyphenols, IL-1β, IL-6	N.S.	Increase	[311]
			Carbonyls, LDH, TNF-α	N.S.	Decrease	[311]
			Blood glucose, Glycosylated hemoglobin, Insulin, FRAP, SOD, GPx, MDA, Aldose reductase, Adiponectin, Ox-LDL	N.S.	Unchanged	[311]
Commercial propolis (BioPropolis)	400 mg/day (3 and 6 months)	Propolis: *n* = 24 Placebo: *n* = 26	HbA1c, Plasma glucose, CML,	Decrease	Decrease	[312]

2hp, 2h-postprandial; ALT, alanine aminotransferase; ALP, alkaline phosphatase; AST, aspartate aminotransferase; BMI, body mass index; BUN, blood urea nitrogen; CAT, catalase; CML, N6-(carboxymethyl)-l-lysine; CRP, C-reactive protein; FBG, fasted blood glucose; FRAP, ferric reducing ability of plasma; GPx, glutathione peroxidase; GSH, glutathione; HbA1c, hemoglobin A1c; HDL-C, high density lipoprotein-cholesterol; HOMA, homeostasis model assessment; LDH, lactate dehydrogenase; LDL-C, low density lipoprotein-cholesterol; IL, interleukin; IR, insulin resistance; MDA, malondialdehyde, ox-LDL, oxidized-low density lipoprotein; N.S., data not shown; RLP-C, remnant-like particle-cholesterol; SOD, superoxide dismutase; TAC, total antioxidant capacity; TNF, tumor necrosis factor; Total-C, total cholesterol; VLDL-C, very low-density lipoprotein-cholesterol.
